# Transcription rate strongly affects splicing fidelity and cotranscriptionality in budding yeast

**DOI:** 10.1101/gr.225615.117

**Published:** 2018-02

**Authors:** Vahid Aslanzadeh, Yuanhua Huang, Guido Sanguinetti, Jean D. Beggs

**Affiliations:** 1 Wellcome Centre for Cell Biology, University of Edinburgh, Edinburgh EH9 3BF, United Kingdom;; 2 School of Informatics, University of Edinburgh, Edinburgh EH8 9AB, United Kingdom

## Abstract

The functional consequences of alternative splicing on altering the transcription rate have been the subject of intensive study in mammalian cells but less is known about effects of splicing on changing the transcription rate in yeast. We present several lines of evidence showing that slow RNA polymerase II elongation increases both cotranscriptional splicing and splicing efficiency and that faster elongation reduces cotranscriptional splicing and splicing efficiency in budding yeast, suggesting that splicing is more efficient when cotranscriptional. Moreover, we demonstrate that altering the RNA polymerase II elongation rate in either direction compromises splicing fidelity, and we reveal that splicing fidelity depends largely on intron length together with secondary structure and splice site score. These effects are notably stronger for the highly expressed ribosomal protein coding transcripts. We propose that transcription by RNA polymerase II is tuned to optimize the efficiency and accuracy of ribosomal protein gene expression, while allowing flexibility in splice site choice with the nonribosomal protein transcripts.

Splicing is the process of removing introns from precursor messenger RNAs (pre-mRNAs) and joining adjacent exons to produce spliced mRNA. The excised intron, in the form of a branched lariat, is rapidly debranched and discarded. If genes contain multiple introns, alternative splicing pathways can give rise to distinct mRNA and protein isoforms by using alternative splice sites or by including or excluding particular exons. In human cells, ∼95% of transcripts are alternatively spliced, thereby greatly expanding the coding capacity of the genome (Kornblihtt et al. 2013). Moreover, alternative splicing events that introduce translational stop codons in mRNAs are generally coupled with nonsense-mediated decay (NMD) to down-regulate that spliced isoform, offering an additional layer of gene expression regulation (Lewis et al. 2003; Sayani et al. 2008; Kawashima et al. 2014). In *Saccharomyces cerevisiae* (budding yeast) only ∼5% of genes contain an intron, although they produce ∼27% of total mRNA, because many intron-containing genes are highly expressed (Ares et al. 1999). There is extensive evidence that in both metazoans and budding yeast, the process of splicing occurs as soon as the intron is transcribed and before transcription termination, that is, cotranscriptionally (Alexander et al. 2010b; Ameur et al. 2011; Carrillo Oesterreich et al. 2016). As a result of splicing being cotranscriptional, RNA polymerase II (RNAPII) elongation rate can influence splicing. According to one model, referred to as the “kinetic coupling” model, variations in RNAPII elongation rate can alter the time available, or the “window of opportunity” for upstream splice sites to be recognized before competing downstream splice sites are produced (Roberts et al. 1998; de la Mata et al. 2003; Kornblihtt 2007; Naftelberg et al. 2015; Saldi et al. 2016). Consistent with this model, it was shown that the RNAPII elongation rate is tuned by different elongation factors and other barriers like chromatin structure, which thereby impact splicing decisions (Kornblihtt 2007; Naftelberg et al. 2015; Saldi et al. 2016). The coupling process is not unidirectional; splicing can also modulate transcription elongation (Brinster et al. 1988; Fong and Zhou 2001; Furger et al. 2002; Kornblihtt et al. 2004; Alexander et al. 2010b).

Transcription elongation mutants that transcribe faster or slower than normal, or chemical inhibitors that reduce the rate of elongation, have been used to study the importance of kinetic coupling and its consequences for splicing outcome (de la Mata et al. 2003; Howe et al. 2003; Ip et al. 2011; Braberg et al. 2013). Studies with mammalian cells have shown that changes in elongation rate impact upstream versus downstream alternative splice site selection and exon inclusion versus exclusion rates in an exon- or intron-specific manner that is not readily predictable. In cultured human cells, slow RNAPII was observed to promote inclusion of the alternatively spliced fibronectin EDI exon (de la Mata et al. 2003) but caused skipping of exon 9 in a CFTR minigene transcript due to more effective recruitment of a negative splicing factor to the 3′ splice site (SS) of the affected exon (Dujardin et al. 2014). Indeed, a genome-wide study of alternative splicing in human cells revealed that slow elongation can disrupt the rate of cassette exon inclusion and exclusion similarly (Fong et al. 2014). Therefore, altering transcription elongation rate can adjust the time allowed and/or RNA accessibility for effective recruitment of splicing regulatory factors.

According to the “recruitment coupling” model, the local concentration of splicing regulatory factors in the proximity of the pre-mRNA can increase as a consequence of their dynamic association with the transcription machinery or chromatin, thereby modulating splicing (Monsalve et al. 2000; de la Mata and Kornblihtt 2006; Das et al. 2007; Luco et al. 2010; Huang et al. 2012; Dujardin et al. 2014).

Although alternative splicing is a relatively rare event in budding yeast, it is important for the regulated expression of some genes. For example, intron retention in *PTC7* transcripts and alternative splicing of an intron at the 3′ end of *FES1* create mRNAs that code for different protein isoforms (Juneau et al. 2009; Gowda et al. 2016). Also, alternative 5′SS use in *SRC1* transcripts results in different cellular localization of the Src1 protein isoforms, and expression of the *APE2* gene is regulated upon heat shock by alternative 3′SS use (Meyer et al. 2011; Mishra et al. 2011). As alternative splicing requires flexibility in splice site use, not all splice sites have consensus sequences. Splicing quality control mechanisms are thought to monitor the configuration of substrate RNAs in splicing complexes to determine whether appropriate splice site sequences have been selected (Semlow and Staley 2012). As most splicing occurs cotranscriptionally, quality control mechanisms should also function cotranscriptionally, raising questions as to whether aspects of transcription could affect splicing quality control.

Here, we investigate the effect of different transcription elongation speeds on various splicing characteristics in *S. cerevisiae*. Following very brief metabolic labeling of RNA with 4-thiouracil (4tU), we isolate newly synthesized RNA (Barrass et al. 2015) and monitor transcription and splicing at high temporal resolution in yeast cells with fast and slow RNAPII mutants. We also assess cotranscriptional splicing by measuring coprecipitation of the spliced mRNA and excised intron that are associated with RNAPII. By using deep RNA sequencing, we determine for the first time how transcription elongation mutants affect splicing fidelity and alternative splicing in budding yeast.

## Results

### Altering transcription elongation rate changes the amount of nascent RNA that is spliced cotranscriptionally

To gain further insight into the coupling between splicing and transcription, we examined the splicing of newly synthesized transcripts produced by RNAPII elongation mutants that transcribe faster (*rpb1-G1097D*) or slower (*rpb1-H1085Y*) than the wild type (WT; *RPB1*) (Kaplan et al. 2012). The mutations reside in the trigger loop of the large subunit, a mobile domain that is responsible for substrate selection and catalysis. The *rpb1-G1097D* allele has been determined to result in approximately four times faster elongation in vitro, whereas the *rpb1-H1085Y* allele results in about eight times slower elongation in vitro, and both mutant alleles reduce the growth rate compared to WT (Supplemental Fig. S1; Kaplan et al. 2012).

**Figure 1.**
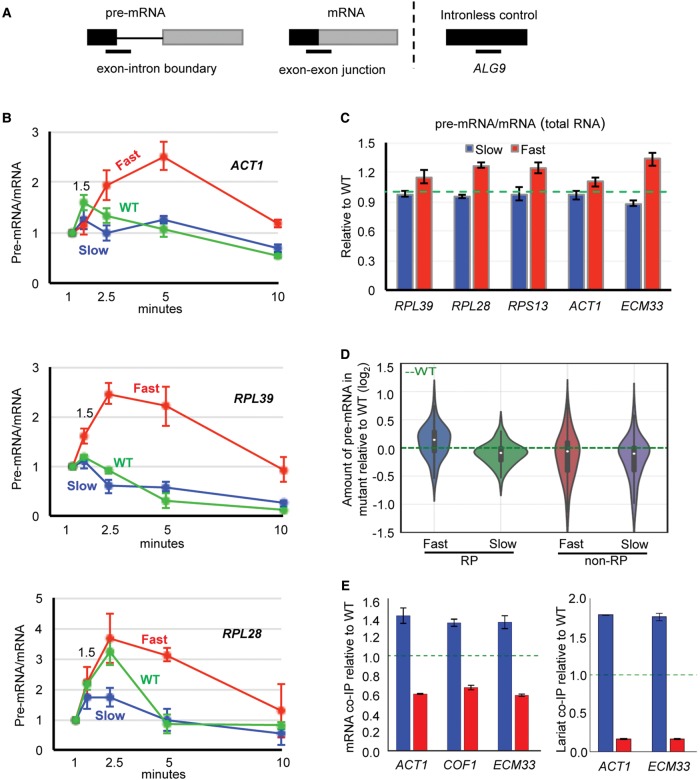
Fast elongation reduces and slow elongation enhances cotranscriptional splicing. (*A*) Diagram showing the location of RT-qPCR amplicons (lines *below*) for measuring pre-mRNA (exon–intron boundary at the 5′SS) and mRNA (exon–exon junction) levels. Black and gray boxes represent exons of intron-containing transcript or coding sequence of intron-less transcript (*ALG9*). (*B*) RT-qPCR results showing pre-mRNA/mRNA ratio for *ACT1*, *RPL39*, and *RPL28* in fast (red), slow (blue), and WT (green) strains. To correct for amount of input RNA, values for pre-mRNA and mRNA were separately normalized to an intron-less transcript (*ALG9*) in the same sample. 4tU labeling was performed for 1, 1.5, 2.5, 5, and 10 min (*x*-axis), with all values plotted relative to the 1-min value. (*C*) Pre-mRNA/mRNA ratio of steady-state RNA for fast and slow mutants relative to WT (green dotted line). Error bars, at least three biological replicates. (*D*) Amount of pre-mRNA in the fast and the slow mutant relative to WT for RP and non-RP transcripts, measured by DICEseq (Huang and Sanguinetti 2016) from RNA sequencing data. (*E*) Fold enrichment of mRNA (*left*) and lariat-intron (*right*) association with RNAPII, in slow (blue) or fast (red) mutants relative to WT (green dotted line), measured by RT-qPCR. To correct for differences in the amount of RNA pull down, values for mRNA and lariat were separately normalized to RT-qPCR values for an intron-less transcript (*ALG9*). Error bars, three biological replicates.

Splicing efficiency is generally determined by measuring levels of spliced and unspliced RNAs at steady state (Braberg et al. 2013). However, the level of these RNA species is determined by the rates of transcription and degradation as well by the efficiency of splicing. By briefly labeling RNA with 4tU, we could affinity-select nascent, thiolated RNA using streptavidin beads. This method enables capture of the majority of short-lived RNA species, including unspliced pre-mRNAs before they are degraded (Barrass et al. 2015). By analyzing the nascent RNA by reverse transcription-quantitative PCR (RT-qPCR), we followed the kinetics of splicing for three well-expressed intron-containing genes: *RPL39*, *RPL28*, and *ACT1* (Fig. 1A). In the fast mutant, we observe an initial rapid increase in the pre-mRNA/mRNA ratio, indicating that pre-mRNA was synthesized faster than it was removed by splicing (Fig. 1B). After 2.5 min, this ratio declined gradually toward the steady-state level. Conversely, with the slow mutant, transcripts were spliced before they accumulated to a significant level such that the pre-mRNA/mRNA ratio was initially less than for the WT RNAPII even in the case of *RPL28* transcripts that splice slowly (Barrass et al. 2015).

We also measured the pre-mRNA/mRNA ratio by RT-qPCR of several intron-containing transcripts in total (steady-state) RNA, again finding a higher ratio than WT (indicating intron retention) with the fast mutant and lower for the slow mutant (Fig. 1C). Next, we performed RNA sequencing and measured steady-state splicing efficiency transcriptome-wide. We observe that the effect of the fast mutant to reduce splicing efficiency (more unspliced pre-mRNA) affects mainly ribosomal protein (RP) transcripts, whereas the slow mutant improves splicing efficiency for both RP and non-RP transcripts (Fig. 1D). By reanalyzing published data (Braberg et al. 2013) from a splicing-specific microarray analysis of steady-state RNA from the same fast RNAPII mutant as used here and a different slow mutant, we found the same effect (Supplemental Fig. S2A). Moreover, based on our RNA-seq data, the levels of pre-mRNAs and mRNAs in the RNAPII mutants reveal a negative correlation for splicing, with the fast mutant reducing mRNA levels and the slow mutant increasing mRNA levels (Supplemental Fig. S2B,C).

The highly stable association of nascent transcripts with elongating RNAPII permits cotranscriptional splicing to be measured by analysis of transcripts that copurify with RNAPII (Churchman and Weissman 2011). For this purpose, the TAP-tagged Rpb3 subunit of RNAPII was pulled down, and copurified RNAs were analyzed by RT-qPCR to quantify the mRNA and lariat-intron–containing products of splicing. Our results reveal increased association of the mRNA and lariat-intron with slow RNAPII, indicating enhanced cotranscriptional splicing, and less association with the fast RNAPII compared with WT (Fig. 1E). This is compatible with the 4tU analysis (Fig. 1B).

### Altering RNAPII elongation rate affects splicing fidelity

**Figure 2.**
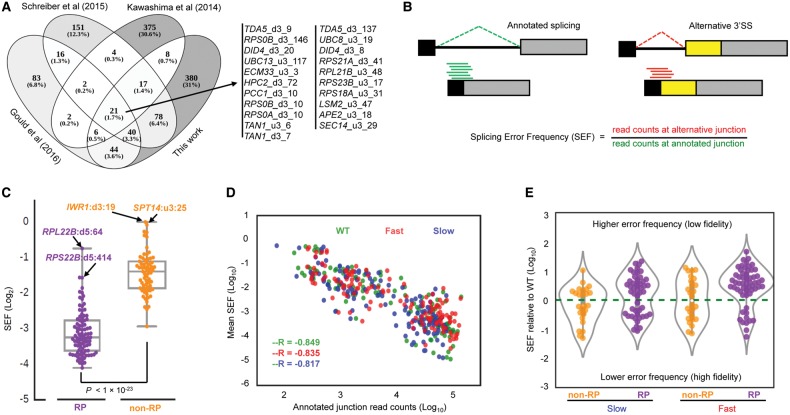
Changes in splicing error frequency (SEF) in fast and slow strains. (*A*) Overlap between novel splicing events found in this work with those in recent reports. We do not detect some of the events reported in the other studies most likely because we filter out events with fewer than five supporting reads (see text). Kawashima et al. (2014) reported any event detected with one or more supporting read. Schreiber et al. (2015) reported events supported by at least three reads. Gould et al. (2016) calculated an entropy value for each junction and reported all the events with entropy ≥2 bits. (*B*) Diagram showing how SEF of novel splicing events was measured using an alternative upstream 3′SS event as an example. (*C*) Distribution of the SEF in RP (purple) and non-RP (orange) intron-containing transcripts in the WT strain. Events with high SEF are highlighted (u3 and d3 are alternative upstream and downstream 3′SS, and d5 is alternative downstream 5′SS; numbers indicate the distance in nucleotides). The *P*-value was obtained by *t*-test. (*D*) Negative correlation between mRNA (both RP and non-RP) abundance and average SEF in fast (red), slow (blue), and WT (green). mRNA abundance was estimated from the number of reads aligned to the exon–exon junctions. –R is Pearson's correlation coefficient. *P*-values for each strain were as follows: fast, *P* < 1 × 10^−33^; slow, *P* < 1 × 10^−31^; and WT, *P* < 1 × 10^−38^. (*E*) Violin plot showing distribution of SEF of non-RP and RP intron-containing transcripts in fast and slow mutants normalized to WT. This plot includes all novel splicing events whose SEF was significantly different in mutants relative to WT (Fisher's exact test; *P* < 0.01, FDR < 0.03). Points *above* dashed line (zero) are novel events with higher SEF than WT (reduced fidelity); points *below* dashed line are novel events with lower SEF than WT (improved splicing fidelity).

To examine whether RNAPII mutants that alter elongation rate affect splicing fidelity, we carried out strand-specific deep sequencing of total RNA from strains in which *UPF1* was deleted in order to protect misspliced transcripts from NMD. After aligning the reads to the genome, we obtained, on average, 5,804,817 uniquely mapped split reads per sample, supporting about 3358 potential splicing events. Events that were unlikely to be products of spliceosome activity, such as with mitochondrial transcripts, and events with fewer than five supporting reads (in at least one of the samples) were excluded from further analysis. The remaining exon–exon splice junctions were divided into two groups, known and novel. The known junctions are those that are annotated in the GTF file (Ensembl, version R64-1-1.75) and the novel junctions result from splicing events that are not annotated in the GTF file. Merging the list of novel splicing events found here with those reported in recent transcriptome-wide studies creates a database of alternative and cryptic splicing events for *S. cerevisiae* (Supplemental Table S1). This includes 21 alternative splicing events that have been reported repeatedly (Kawashima et al. 2014; Schreiber et al. 2015; Gould et al. 2016) and hundreds of new splicing events that were not in those data sets (Fig. 2A). In order to ensure robustness of the splicing fidelity analysis, we performed further filtering, selecting only events that were supported by at least five reads in both replicates of one or more strains. Finally, 244 novel alternative splicing events were retained, and the splicing error frequency (SEF) was calculated for each event (Fig. 2B; Supplemental Table S1). First, by analyzing novel events occurring in the strain with WT RNAPII, we note a marked difference between RP and non-RP transcripts (Fig. 2C). Despite the greater abundance of RP transcripts, their SEFs (ranging from one in 100,000 up to one in 250) are orders of magnitude lower than those of non-RP transcripts (SEFs ranging from one in 100 to seven in 100). Some novel events were detected with much higher frequency; for example, *IWR1* and *SPT14* could potentially produce similar amounts of their two alternative isoforms (Fig. 2C). The SEF and the mRNA abundance are anti-correlated for both RNAPII mutants and WT (Fig. 2D), indicating that the detection of alternative splicing events in high-depth RNA-seq data is not due to the higher number of reads for highly expressed transcripts. The position distribution of the novel alternative splicing events in the WT strain shows that most of the 166 alternative 3′SSs occur a very short distance from the annotated 3′SS. Although there are only half as many alternative 5′SSs (78 events) (Supplemental Fig. S3), alternative 3′SSs and 5′SSs have similar SEF distributions, and these values are significantly lower for RPs than for non-RPs (boxplot in Supplemental Fig. S3). The majority of the novel alternative splicing events introduced premature translation termination codons in the coding region of the spliced RNAs, which would normally be recognized by the NMD system, leading to transcript degradation (Supplemental Table S1). By using the Fisher's exact test (*P* < 0.01, false-discovery rate [FDR] < 0.03) to determine which novel splicing events occur more or less frequently in the fast mutant relative to WT, we identified 57 in RP transcripts, with an approximately 4:1 ratio of reduced splicing fidelity (higher SEF) over increased fidelity (lower SEF) in the fast mutant (Fig. 2E). In comparison, 26 novel events in non-RP transcripts include approximately equal numbers with reduced and enhanced fidelity. With the slow mutant, we identified 48 novel events in RP transcripts, of which reduced fidelity occurred approximately 1.6 times more often than increased fidelity (Fig. 2E). Similar to the fast mutant, we detected equal numbers of events with reduced and enhanced fidelity with the slow mutant in non-RP transcripts. Collectively, these results demonstrate that fidelity of splicing RP intron-containing transcripts is more sensitive to changes in transcription elongation rate than for non-RP transcripts.

**Figure 3.**
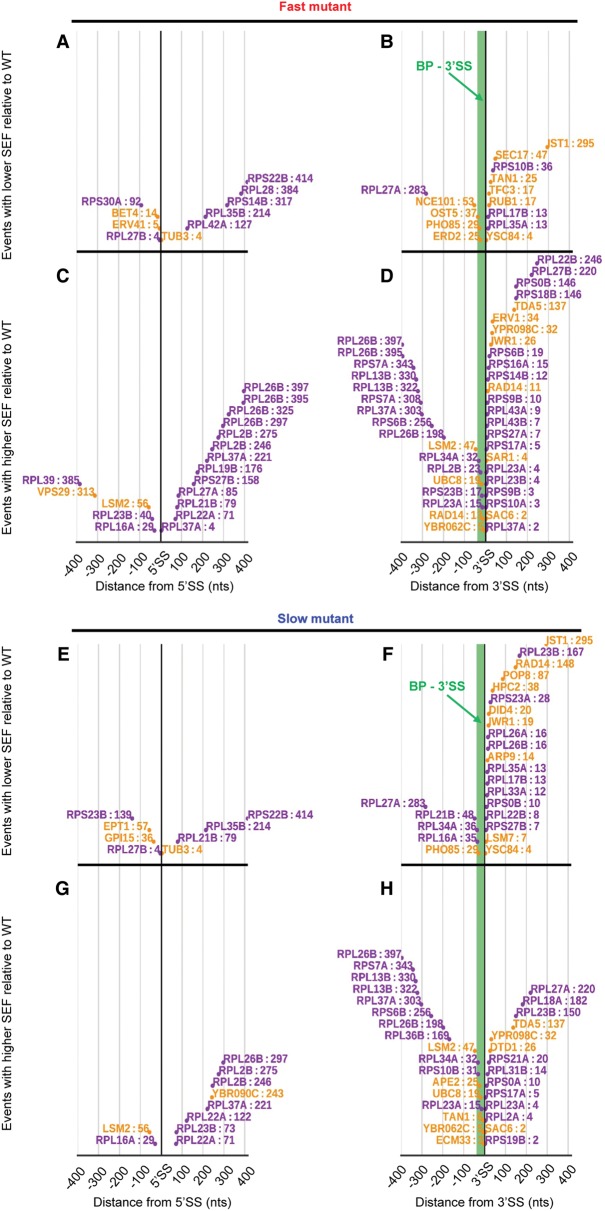
Distribution of the novel splice sites in RP (purple) and non-RP (orange) intron-containing transcripts whose SEF is significantly different in the fast and slow mutants relative to WT (*P* < 0.01). (*A*,*B*) Alternative 5′SS and 3′SS splicing events whose fidelity increased with the fast mutant. (*C*,*D*) Alternative 5′SS and 3′SS splicing events whose fidelity reduced with the fast mutant. (*E*,*F*) Alternative 5′SS and 3′SS splicing events whose fidelity increased with the slow mutant. (*G*,*H*) Alternative 5′SS and 3′SS splicing events whose fidelity reduced with the slow mutant. Green region upstream of 3′SS is the mean distance between BP and annotated 3′SS in budding yeast (∼37 nt).

With WT RNAPII, novel upstream 3′SS use is mainly restricted to within a window of ∼50 nucleotides (nt) upstream of the annotated 3′SSs (Supplemental Fig. S3). Notably, position distribution of the alternative splice sites whose SEF is significantly different in the fast (Fig. 3A–D) and slow (Fig. 3E–H) mutants relative to WT shows increased use of alternative 3′SSs that occur upstream of the annotated branch point (BP) (note the change in slope of the plot of upstream novel 3′SSs in Fig. 3D,H compared with Supplemental Fig. S3). These events likely make use of more 5′ suboptimal BPs for splicing. For example, two novel 3′SS events in *RPL13B* are located 330 and 322 nt upstream of the annotated 3′SS, and lariat sequencing (Gould et al. 2016) has detected a cryptic BP (AACTAAT) located 21 and 13 nt upstream of these cryptic 3′SSs, respectively (Supplemental Fig. S4). The SEFs for these novel events in *RPL13B*, and similar events in other transcripts, were increased by both faster and slower transcription, most likely due to increased utilization of more 5′ cryptic BPs (Fig. 3D,H). Notably, fast and slow transcription do not necessarily reduce or increase a specific splicing event in opposite directions. For instance, an alternative downstream 5′SS in *RPS27B* increases with faster transcription. However, both the fast and slow mutants increase use of an alternative upstream 3′SS in *RPL26B* (Supplemental Fig. S4). The elongation mutants do not affect occurrence of some alternative splicing events; for example, alternative 3′SSs in *SEC14* and *UBC12* are used with the same frequency in all three strains (*P* > 0.05) (Supplemental Fig. S4). We validated several of these alternative and novel splicing events by RT-PCR (Supplemental Fig. S5).

### Features associated with splicing fidelity

**Figure 4.**
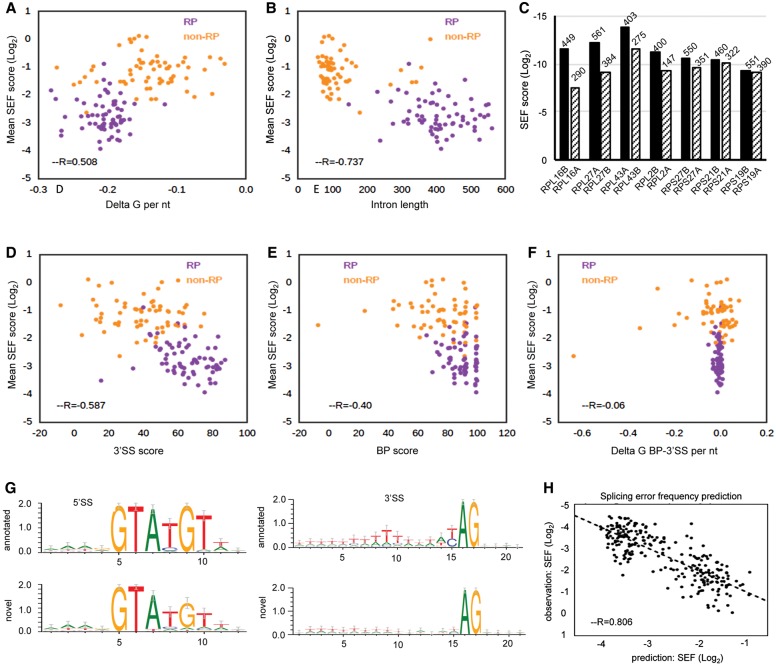
Features associated with SEF. Orange points represent novel events in non-RP transcripts, and purple points denote RP transcripts. (*A*) Positive correlation between delta G of intron (see Methods) and mean SEF of the transcripts. Delta G of intron was divided by the intron length to achieve delta G per nucleotide. More negative delta G values represent more structured introns. (*B*) Negative correlation between intron length and mean SEF. (*C*) Mean SEF of seven pairs of paralogs. Paralogs with shorter intron length (IL) have higher SEF. (*D*) Negative correlation between 3′SS score (see Methods) and mean SEF. The score expresses how similar the splice sites are to the budding yeast 3′SS consensus. (*E*) Weak negative correlation between BP score and mean SEF. (*F*) Absence of correlation between delta G of BP-3′SS region and mean SEF. Delta G was divided by the distance between BP and 3′SS to achieve delta G per nucleotide (*G*) Sequence logos were generated for 5′SSs and 3′SSs of all novel alternative splicing events. For annotated introns, sequence logos were generated only from splice sites of transcripts that had novel alternative splicing events. (*H*) Correlation between observed and predicted SEF (as described in Methods).

In order to identify features that might be responsible for modulating splicing fidelity, we investigated the correlation between splicing error rate in all three strains and various transcript features, including intron length, predicted intron secondary structure (free folding energy, normalized by the intron length), 5′SS, BP and 3′SS scores, and frequency of 1- to 3-mers in the intron (Supplemental Fig. S6A). Transcripts with introns predicted to have less stable structure (higher free folding energy) have higher SEF (*P* < 1 × 10^−10^) (Fig. 4A). There is also a significant negative correlation between the intron length and SEF, indicating that shorter introns are more susceptible to splicing errors (*P* < 1 × 10^−23^) (Fig. 4B). This leads to the prediction that within pairs of RP paralogs that have introns of different lengths (but very similar or identical exons), the one with a shorter intron will have higher SEF than the other. Indeed, this is the case for seven RP paralogs whose intron lengths differ by >100 nt (Fig. 4C; Supplemental Fig. S7A). A similar comparison of seven RP paralogs whose introns differ by <34 nt found no significant difference in their SEF (Supplemental Fig. S7B). As might be predicted, 3′SS score anti-correlates with splicing error rate (*P* < 1 × 10^−13^) (Fig. 4D). Notably, the effect on splicing error rate of having a poor 3′SS score is also significantly greater within RP genes (*P* < 0.05) (Fig. 4D). There is also a weak but significant negative correlation (*P* < 1 × 10^−6^) between BP score and mean SEF, indicating a small contribution of the BP in maintaining splicing fidelity (Fig. 4E). However, delta G of the BP-3′SS region and splicing fidelity show no correlation (*P* > 0.05) (Fig. 4F). Collectively, greater SEF (lower fidelity) correlates with shorter introns together with less stable intron secondary structure and poor 3′SS score. We also generated sequence logos for 5′SSs and 3′SSs of novel introns, finding that the novel introns predominantly have suboptimal splice sites (Fig. 4G). The polypyrimidine tract preceding the novel 3′SSs also shows a relaxation from the consensus sequence, highlighting the significance of the polypyrimidine tract sequence in correct recognition of the 3′SS, even in budding yeast (Plass et al. 2012). Additionally, our analysis shows that novel RP introns whose use increases with fast or slow mutants have significantly higher predicted free energy (Supplemental Fig. S7C,D). In contrast, novel events that are less frequent (increased fidelity) with the RNAPII mutants do not have different free energy compared with annotated introns. This suggests that selection of novel introns with less stable secondary structure increases with changing transcription elongation rate. Using these and other features listed in Supplemental Figure S6A to predict the splicing error rate by a random forest regression model reveals a good correlation (R = 0.804) between observed and predicted splicing error rate (Fig. 4H).

### Altering transcription speed increases splicing of cryptic introns and enhances exon skipping

**Figure 5.**
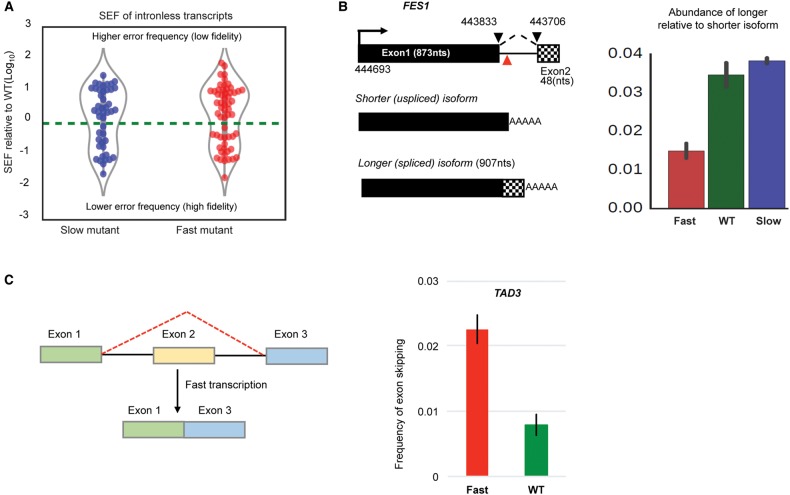
(*A*) Violin plots show the occurrence of cryptic splicing events within intron-less transcripts in the slow and fast mutants relative to WT. This plot includes all novel splicing events with significantly different SEF in mutants relative to WT (Fisher's exact test; *P* < 0.01). Points *above* the dashed line (zero) are splicing events with SEF greater than WT (reduced fidelity). Points *below* the dashed line are the events with improved splicing fidelity compared with WT. (*B*) Splicing of *FES1* intron. Intron (ends connected by dashed lines) is 128 nt, and red triangle sign shows where promotor proximal polyadenylation occurs. Splicing of the intron generates a longer isoform. Barplot shows relative abundance of the longer (spliced) isoform relative to shorter isoform, measured by dividing junction read counts of longer isoform over FPKM of the shorter isoform. (*C*) Second exon skipping in *TAD3* transcript with the fast mutant. To calculate frequency of exon skipping, the number of exon1–exon3 junction (exon skipping) reads was divided by the number of exon1–exon2 junction (first intron splicing) reads. A similar result is obtained by dividing by the number of exon2–exon3 junction reads.

The results described above refer to errors in splicing transcripts from the ∼5% of genes that are known to contain introns. Due to the depth of the RNA sequencing, splicing of cryptic introns was detected for 140 normally unspliced transcripts, as evidenced by at least five unique splice junction reads in the WT sequence data (Supplemental Table S1). Perturbing RNAPII elongation rate in either direction increases splicing of these cryptic introns compared with WT (Fig. 5A). This is presumably a consequence of fast and slow transcription reducing splicing fidelity. Interestingly, splicing of the intron at the 3′ end of *FES1* (Gowda et al. 2016) is affected differentially with elongation rate. With the fast mutant, a twofold increase in the *FES1* shorter isoform that results from using the promoter-proximal polyadenylation site and a slight increase in splicing of the intron (generating longer isoform) with the slow mutant suggest cotranscriptional regulation of *FES1* splicing (Fig. 5B; Supplemental Fig. S7E). This result does not conform with a simple kinetic model that would predict that faster elongation might increase 3′SS recognition and enhance splicing. However, as we have shown that fast transcription decreases cotranscriptional splicing, this result may be explained if cotranscriptional cleavage and polyadenylation is less affected by faster elongation. This is consistent with preferential induction of the shorter isoform due to increased transcription upon heat shock (Gowda et al. 2016).

However, splicing of some cryptic introns found with WT RNAPII appear insensitive to alteration in transcription speed, for example, *PRP5* and *MRM2* that have 167- and 63-nt introns, respectively, with canonical splice sites (Miura et al. 2006; Yassour et al. 2009). Previously, only *RPL30* was reported to have a novel intron in the second exon (Schreiber et al. 2015). Here we report five more genes like this (Supplemental Table S1). As with other splicing events that disrupt translational open reading frames, such nonproductive splicing may provide a mechanism to reduce expression of these genes by promoting the turnover of the spliced RNA, referred to as spliceosome-mediated decay (Volanakis et al. 2013). Recently, Gould et al. (2016) reported finding an unusual (for yeast) AT-AC intron nested inside the annotated intron of *RPL30*, but neither we nor two other studies (Kawashima et al. 2014; Schreiber et al. 2015) found evidence for this.

Only 10 genes in *S. cerevisiae* contain two introns, and RT-PCR evidence of exon skipping was reported for few of these (Hossain et al. 2011; Egecioglu et al. 2012). Searching for evidence of exon skipping in our RNA-seq data, we found a threefold increase in skipping of the *TAD3* second exon in the fast mutant (Fig. 5C). Exon skipping is detected with low frequency in other two-intron transcripts, including *DYN2* (Howe et al. 2003) but with insufficient read density to draw a clear conclusion (Supplemental Fig. S8). There is no sign of exon skipping for the highly expressed *RPL7A* and *RPL7B*, the only RP transcripts with three exons, which is consistent with higher splicing fidelity in RP transcripts (Fig. 2C).

## Discussion

Our analysis of the kinetics of splicing nascent 4tU-labeled RNA detects production of the pre-mRNA before it is spliced (Fig. 1). Greater accumulation of the pre-mRNA with the fast RNAPII compared with the WT strain indicates that splicing does not keep up with a faster elongating RNAPII. This is in accord with results from single-molecule intron tracking (SMIT), which measures the distance that RNAPII has transcribed past the intron when cotranscriptional splicing occurs (Carrillo Oesterreich et al. 2016). SMIT showed that for 87 genes tested, the fast RNAPII transcribed significantly further than normal RNAPII when splicing occurred, but they did not test a slow mutant (Carrillo Oesterreich et al. 2016). Our data indicate that splicing occurs sooner after transcription by slower RNAPII compared with WT RNAPII. In support of this, we show more spliced mRNA associated with slow RNAPII and less with fast RNAPII compared with WT. Together, these different assays show that with faster elongation, less splicing occurs cotranscriptionally but more of the second exon may be available to the splicing machinery at the time when splicing takes place, whereas with slower elongation, more splicing takes place cotranscriptionally and sooner after transcription of the 3′SS. Importantly, these results suggest that when transcription elongation rate changes, the rate of splicing does not change correspondingly. This is consistent with the window of opportunity model, which proposes that slow elongation expands and fast elongation compresses the time available for upstream splice sites to be recognized before competing downstream splice sites are produced (Kadener et al. 2001; de la Mata et al. 2003; Kornblihtt 2007; Saldi et al. 2016). In the case of single intron genes, this translates to more or less time being available for splicing to occur before 3′ end processing occurs and transcription terminates, that is, cotranscriptionally.

Our measurements of splicing efficiency, either transcriptome-wide by RNA-seq or by RT-qPCR of total RNA, reveal an inverse correlation between elongation rate and splicing efficiency (Fig. 1; Supplemental Fig. S2). A previous study with RNAPII elongation mutants also documented an anti-correlation between splicing efficiency and transcription elongation speed in budding yeast based on the ratio of intron to spliced exon junction signals from microarray data (Braberg et al. 2013). We reveal that the overall reduced splicing efficiency in the fast mutant is due to an effect predominantly on RP transcripts, whereas the slow mutant improves splicing efficiency for both RP and non-RP transcripts (Fig. 1). Moreover, this is not simply due to there being more or less unspliced pre-mRNA as a consequence of different decay rates in the mutants, as the level of spliced mRNA is elevated in the slow mutant and reduced in the fast mutant compared with the WT (Supplemental Fig. S2B,C). In the case of slower elongation, the increased splicing efficiency may be explained by splicing factors having an increased opportunity to bind to splice sites as they emerge from the RNAPII exit channel before other competing events occur downstream. The corollary is that in the fast mutant the lower splicing efficiency could be due to the greater potential for competing events, such as RNA secondary structure that masks splice sites (Eperon et al. 1988), or to competition with downstream cryptic splice sites (discussed below). As there is less spliced mRNA at steady state with the fast RNAPII, it appears that post-transcriptional splicing does not compensate for the reduced amount of cotranscriptional splicing. This is an important observation, as it implies that it is advantageous to splice cotranscriptionally.

We also tested, for the first time, how RNAPII elongation mutants affect alternative splicing or splicing fidelity (annotated versus novel splicing events) transcriptome-wide in budding yeast (illustrated Fig. 2B). We find that, with WT RNAPII, RP transcripts in general are spliced with higher fidelity than non-RP transcripts (Fig. 2C), and splicing fidelity correlates with mRNA abundance (Fig. 2D), 3′SS score, and longer and more highly structured introns (Fig. 4); all features that are typical of RP introns. Nevertheless, we show that splicing fidelity of RP transcripts is reduced by changes in transcription speed, especially by faster transcription, whereas non-RP introns are less sensitive to changes in elongation rate, with fidelity being moderately increased or reduced with the same likelihood (Fig. 2E). We conclude that, especially for RP transcripts, speed of transcription is critical for both the efficiency and the fidelity of splicing.

With WT RNAPII, more novel 3′SSs are used than novel 5′SSs, and it is the frequency with which these novel splice sites are used, rather than the number of different types of splicing events, that is lower for RP compared with non-RP transcripts. Meyer et al. (2011) reported that real (canonical) 3′SSs are more accessible than are cryptic 3′SSs due to effects of intron structure, which might explain why cryptic splice sites are less frequently used in RP transcripts that, in general, have more highly structured introns. Non-RP introns, on average, have lower 3′SS scores than RP introns (Fig. 4D), which may contribute to their higher error frequency. Moreover, the productive alternative splicing events identified to date in budding yeast are exclusively in non-RP transcripts, reflecting the need for greater flexibility in splice site choice.

The higher SEF for RP transcripts with the RNAPII mutants is due mainly to an increased error frequency for the same novel splice sites found with WT RNAPII, rather than use of new splice sites in the mutants (Fig. 3). Also, more errors occur in 3′SS selection with RP transcripts (Fig. 3); therefore although 3′SS score correlates with fidelity in yeast, this does not seem to protect against the effects of changing elongation rate, at least for the RP transcripts. This is unlike the situation with human fibronectin exon domain I (EDI), where responsiveness of exon skipping to elongation rate was inversely proportional to 3′SS strength (Nogués et al. 2003).

It has been proposed that spliceosomes can use any 3′SS located within an optimal distance (10–45 nt) downstream from the BP, but RNA structures can make optimal 3′SSs more accessible while masking suboptimal 3′SSs (Meyer et al. 2011). For example, there is a cryptic 3′SS between BP and annotated 3′SS of *RPS23B*, but it is masked by secondary structures (Meyer et al. 2011). Interestingly, the fast mutant enhances use of this cryptic 3′SS, suggesting that faster transcription weakens the structure of pre-mRNA in this region, making the suboptimal 3′SS more accessible (Supplemental Fig. S4G). In contrast, the elongation mutants do not increase SEF of an alternative upstream 3′SS event in *APE2* (Supplemental Fig. S4H), which was shown to be spliced with higher frequency upon heat shock, probably because of unstable RNA structure in the BP-3′SS region at elevated temperature (Yassour et al. 2009; Meyer et al. 2011). These different effects of transcription elongation on cryptic 3′SS use in *RPS23B* and *APE2* might suggest that changes in elongation rate preferentially affect stable RNA structure of the RP transcripts, which is consistent with the observed correlation between intron structure and splicing fidelity (Fig. 4A). However, we did not observe a genome-wide correlation between SEF and delta G of BP-3′SS regions (Fig. 4F). Conceivably, BP-3′SS structure affects use of canonical 3′SSs in a gene-specific manner, and a more detailed analysis is required to investigate these effects.

Downstream novel 3′SSs mainly occur very close to the annotated 3′SSs (Supplemental Fig. S3), presumably constrained by the 10- to 45-nt optimal distance from the BP (Meyer et al. 2011) rather than any lack of downstream “AG” dinucleotides (potential 3′SSs) (Supplemental Fig. S6B). Those few novel 3′SSs that occur further downstream could use cryptic BPs. For example, lariat sequencing detected two additional BPs in *TDA5* downstream from the annotated BP (Gould et al. 2016) that could explain our finding of a suboptimal 3′SS 127 nt downstream from the annotated 3′SS in *TDA5* (Supplemental Fig. S4A).

Howe et al. (2003) showed that mutations that create a suboptimal BP sequence in the first intron of *DYN2* caused second exon skipping and that the defect was partially alleviated by slower transcription. This suggests that slow transcription provides more time for the assembling spliceosome to select the suboptimal BP (within the first intron) before transcription of the competing downstream BP (within the second intron). However, our results show that both faster and slower transcription reduces BP fidelity, with increased utilization of suboptimal BPs upstream of the annotated BP, leading to splicing of proximal cryptic 3′SSs (Fig. 3D,H; Supplemental Fig. S4).

How does transcription elongation affect splicing fidelity? The effects on splicing fidelity of changing transcription rate could reflect less efficient elimination of splicing errors as a result of perturbing cotranscriptional quality control mechanisms, such as kinetic proofreading of splicing (Hopfield 1974; Semlow and Staley 2012). Additionally, altering transcription elongation rate was reported to change multiple properties of nascent RNA and the chromatin environment around sites of active transcription, affecting alternative splicing decisions (Buratti and Baralle 2004; Dujardin et al. 2013; Saldi et al. 2016). The CTD was recently shown to form multivalent interactions with low-complexity domains in other proteins, including certain splicing factors, suggesting another mechanism by which RNAPII might affect cotranscriptional splicing, by compartmentalizing these two processes (Harlen and Churchman 2017). Therefore, a finely tuned coordination in time and space between transcription and splicing may be influenced by many factors.

The relationship that we observe between transcription elongation rate and the use of alternative/cryptic splice sites is not readily explained by a simple “window of opportunity” model, as both faster and slower transcription reduce splicing fidelity. This is more in keeping with the “Goldilocks” model in which an optimal elongation rate is required for a normal processing outcome (Fong et al., 2014). We propose that RNAPII speed may be tuned to optimize splicing efficiency and to balance fidelity with flexibility for making alternative splice site choices in yeast, as in mammals. Studies in budding yeast revealed that RP transcripts tend to be spliced faster and more cotranscriptionally than non-RP transcripts (Barrass et al. 2015; Wallace and Beggs 2017). Our data now show that the efficiency and fidelity of splicing RP transcripts are affected by altered transcription rates, especially by faster elongation, significantly more than for non-RP transcripts. Together, these observations suggest that splicing of RP transcripts is more functionally coupled to transcription than is splicing of non-RP transcripts, and may indicate that it is beneficial to splice cotranscriptionally. RPs are abundant and vital components of the translation machinery, and RP gene expression is coordinated to match cell growth rate according to the requirement for more or fewer ribosomes in rapidly or slowly dividing cells respectively (Warner 1999; Pleiss et al. 2007). Overall, the transcription and splicing of RP transcripts seem to be tuned to meet the demands of high expression without compromising quantity and quality of the spliced transcripts. It was previously proposed that there is an optimal transcription rate for splicing different introns in mammalian cells (Fong et al. 2014). In view of the greater sensitivity of RP splicing to transcription speed, we propose that, in budding yeast, the RNAPII elongation rate has evolved in tune with RP transcript splicing to optimize the expression of these highly important genes while allowing greater flexibility in splice site choice for non-RP genes. Important goals for the future will be to determine how the transcription and splicing machineries communicate and how these interactions are regulated to ensure the appropriate outcome.

## Methods

### Yeast strains and plasmids

*RPB1* (CKY690), *rpb1-G1097D* (CKY772), and *rpb1-H1085Y* (CKY691) strains were generously provided by Craig Kaplan (Kaplan et al. 2012). *UPF1* was deleted from these strains as previously described (Longtine et al. 1998), selecting *upf1::HPHMX6* transformants on hygromycin B plates (Supplemental Table S2). For 4tU labeling, strains contained pRS426–*FUI1* that encodes the uracil permease Fui1 to boost 4tU uptake by cells.

### RNA extraction

For total RNA, log phase cells were pelleted and lysed in a beadbeater (BioSpec Products) with zirconia beads (Thistle Scientific) and phenol, and then extracted with phenol:chloroform and precipitated with ethanol. For nascent RNA, 4tU was added (final concentration 100 µM) to log phase culture at OD600 of 0.8. After 1, 1.5, 2.5, 5, and 10 min, samples were snap frozen by pouring into chilled methanol sitting on dry ice, and then 4tU-labeled RNA was purified as previously described (Barrass et al. 2015). The yield of 4tU-labeled RNA was similar for all three strains (Supplemental Fig. S9), as estimated by physical measurements (NanoDrop and Agilent Bioanalyzer). For RT-qPCR analysis, equal amounts of nascent RNA were used based on incorporated 4tU. To further correct for amount of input RNA, values for pre-mRNA and mRNA were separately normalized to an intron-less transcript (*ALG9*) in the same sample.

### Native elongating transcript purification

Immunoprecipitation of RNAPII and purification of nascent RNA was performed essentially as previously described (Churchman and Weissman 2001). RNAPII was bound via TAP-tagged Rpb3 to IgG-Sepharose beads, eluted by incubation with ProTEV protease (Promega) for 90 min at 18°C, and then collected by Promega spin column. After proteinase K digestion, RNA was precipitated in ethanol.

### RT-qPCR

RT-qPCR was carried out as described previously (Alexander et al. 2010a) using oligonucleotides listed in Supplemental Table S3.

### RNA sequencing

Total RNA was extracted from log phase culture grown in YPDA and quality controlled by Agilent 2100 bioanalyzer RNA Nano-Chip. BGI (formerly Beijing Genomics Institute) performed the library preparation and strand-specific, 150 base paired-end deep sequencing on an Illumina HiSeq 4000 for two independent biological replicates per strain.

### Processing of the raw sequencing data

RNA-seq reads were mapped to *S. cerevisiae* strain S288C reference genome (version R64) with STAR, a splice-aware aligner (Dobin et al. 2013). To quantify pre-mRNA fractions, the dice-count function from the DICEseq package (Huang and Sanguinetti 2016) was used to obtain the counts for specific read classes; reads that only belong to pre-mRNA are boundary and intron reads, while reads that only belong to mature mRNA are junction reads. We define the pre-mRNA fraction as pre-mRNA reads/(pre-mRNA reads + mRNA reads).

### Estimation of SEF for novel splicing events

Alternative or novel splicing events are defined as splicing events that are within intron-containing transcripts and are not annotated in the *S. cerevisiae* annotation file from Ensembl (version R64-1-1.75). We used default settings of STAR aligner for detecting novel splice junctions. By default, STAR filters all annotated splice junctions with overhang (anchor sequence) length less than three on both sides and requires overhang sequence longer than 12 on both sides for reporting unannotated canonical splice junctions. Events supported by less than five unique reads were filtered out. The frequency of each alternative splicing event is defined as

$$\text{SEF}=\frac{\text{number of reads that align to the novel splice junction}}{\text{number of reads that align to the annotated splice junction}}.$$

### Estimation of SEF for cryptic introns in normally intron-less transcripts

Cryptic introns are those spliced from normally intron-less transcripts and are not annotated in the *Saccharomyces* annotation file. Events supported by less than five unique reads were filtered out. The frequency of splicing cryptic introns is defined as

$$\text{SEF}=\frac{\text{number of reads that align to the cryptic splice junction of the transcript}}{\text{RPKM of the transcript}}.$$

### Sequence features and prediction of SEF

To predict splicing error from sequence, we considered the following features as predictors: intron length, Delta G for intron and for BP-3′SS region, 5′SS score, 3′SS score, BP score, and frequency of 84 short sequences (1- to 3-mers) from both annotated introns and novel introns. For the splice site scores, we examined sequences between 4 nt upstream of and 7 nt downstream from the 5′SS, 16 nt upstream of and 4 nt downstream from the 3′SS, and 7 nt upstream of and 3 nt downstream from the BP. Then the motif scores were measured from the sequences of annotated and alternative introns. The position weight matrix (PWM) was calculated by the empirical frequency of each nucleotide at a certain position; for example, *p*2(*C*) = 0.3 means the weight of C at position 2 is 0.3. By assuming the positions are independent and using the empirical weights for each position, we could define the probability of seeing a certain sequence *S*_*g*_ = {*s*_*g*,1_, …, *s*_*g*,*k*_} on a gene g as

$$P(S_{g})=\overset{k}{\underset{i=1}{\prod }}p_{i}(s_{g,i}).$$

By first applying a log_2_ transformation of the probability *X*_*g*_ = log_2_ (*P*(*S*_*g*_)) and then using a linear re-scale *Y*_*g*_ = (*X*_*g*_ − min(*X*)) / (max(*X*) − min(*X*)) × 100, we have the splice score *Y*_*g*_ for each gene g, ranging from zero to 100 (Crooks et al. 2004). The delta G is a free energy score for RNA secondary structure, which is predicted by mfold v3.6 (Zuker 2003). All these features were fetched with pyseqlib (https://github.com/huangyh09/pyseqlib). In total, 176 features were used to predict the SEF scores for novel splicing events. Based on these features, a random forest regression model was trained to predict the SEF score at log_2_ scale, and threefold cross-validation was used to evaluate the prediction performance.

## Data access

All sequencing data from this study have been submitted to the NCBI Gene Expression Omnibus (GEO; http://www.ncbi.nlm.nih.gov/geo/) under accession number GSE99161.

## Supplementary Material

Supplemental Material

Revised Supplemental Material
