# Supplementary material for: Transcription rate strongly affects splicing fidelity and cotranscriptionality in budding yeast
Source: Genome Res. 2018 Feb;28(2):203–13. doi: 10.1101/gr.225615.117 (PMC5793784; doi:10.1101/gr.225615.117)
Supplement: Supplemental Material [file supp_28_2_203__index.html]

Transcription rate strongly affects splicing fidelity and cotranscriptionality in budding yeast — Supplemental Material 

# Transcription rate strongly affects splicing fidelity and cotranscriptionality in budding yeast

## Supplemental Material

- Supplemental\_Table\_S1.xlsx
- Supplemental\_Table\_S2.docx
- Supplemental\_Table\_S3.docx
- Supplemental\_Figures.pdf
