# Supplementary material for: Transcription rate strongly affects splicing fidelity and cotranscriptionality in budding yeast
Source: Genome Res. 2018 Feb;28(2):203–13. doi: 10.1101/gr.225615.117 (PMC5793784; doi:10.1101/gr.225615.117)
Supplement: Supplemental Material [file supp_gr.225615.117_Supplemental_Table_S2.docx]

**Supplemental Table S2**

Genotype of the strains used in this study:

| *rpb1-G1097D* (CKY772) | *MATa ura3-52 his3∆200 leu2∆1 or ∆0 trp1∆63 met15∆0 lys2-128∂ gal10∆56 rpb1∆::CLONATMX RPB3::TAP::KlacTRP1 ∆upf1::HPH pCK876 rpb1-G1097D (T69 corrected) CEN LEU2* |
| --- | --- |
| *rpb1-H1085Y* (CKY691) | *MATa ura3-52 his3∆200 leu2∆1 or ∆0 trp1∆63 met15∆0 lys2-128∂ gal10∆56 rpb1∆::CLONATMX RPB3::TAP::KlacTRP1 ∆upf1::HPH pCK870 rpb1-H1085Y (T69 corrected) CEN LEU2* |
| *RPB1*-WT (CKY690) | *MATa ura3-52 his3∆200 leu2∆1 or ∆0 trp1∆63 met15∆0 lys2-128∂ gal10∆56 rpb1∆::CLONATMX RPB3::TAP::KlacTRP1 ∆upf1::HPH pCK859 RPB1 (T69 corrected) CEN LEU2* |
