# Supplementary material for: Transcription rate strongly affects splicing fidelity and cotranscriptionality in budding yeast
Source: Genome Res. 2018 Feb;28(2):203–13. doi: 10.1101/gr.225615.117 (PMC5793784; doi:10.1101/gr.225615.117)
Supplement: Supplemental Material [file supp_gr.225615.117_Supplemental_Table_S3.docx]

| *ACT1* pre-mRNA F | TACATCAGCTTTTAGATTTTTCACGCTT |
| --- | --- |
| *ACT1* pre-mRNA R | ATTCTGGTATGTTCTAGCGCTTGCACCATC |
| *ACT1* mRNA F | TCGAAAATTTACTGAATTAACAATGGA |
| *ACT1* mRNA R | GCAAAACCGGCTTTACACAT |
| *ACT1* Lariat F | AGGGGCTTGAAATTTGGAAAAA |
| *ACT1* Lariat R | GCAAGCGCTAGAACATACATAGTACA |
| *RPL39* pre-mRNA F | AACACAGATAGATCAACATGGCTGTATGT |
| *RPL39* pre-mRNA R | GGTGGTAAGGTCATTTAGATGGATGTG |
| *RPL39* mRNA R | GTGGCAATGGTCTGTTTTGCTTC |
| *RPL39* mRNA F | AGATCAACATGGCTGCTCAAAAGTC |
| *RPL28* pre-mRNA F | TCCAGATTCACTAAGACTAGAAAGCACAGA |
| *RPL28* pre-mRNA R | TTGGTTCTTTCATTCCCTCTTCCA |
| *RPL28* mRNA F | TCCAGATTCACTAAGACTAGAAAGCACAGA |
| *RPL28* mRNA R | TGACCACCGGCCATACCTCT |
| *ECM33* pre-mRNA F | AGTGCCTCCGCTCTAGCTGGT |
| *ECM33* pre-mRNA R | CGAGATTTGTGAGGAAAGAGGCAAA |
| *ECM33* mRNA F | GCCTCCGCTCTAGCTGCTAACTC |
| *ECM33* mRNA R | TTGAGCAGTAGCAGTGGCAGAAGT |
| *ECM33* Lariat F | CCTGTCATAGGATTAGGGCGAGT |
| *ECM33* Lariat R | GTATGTACACATTCTCCTTTATAGTATTCCCG |
| *COF1* pre-mRNA F | ATCTGGGTATGCTAAATTTCATTTGTACTCC |
| *COF1* pre-mRNA R | AGCGAGATAAAACAGCATCATGTCAA |
| *COF1* mRNA F | TCTGGTGTTGCTGTTGCTGATG |
| *COF1* mRNA R | CAACGATTTCGGTTTTAGCATCG |
| *RPS13* pre-mRNA F | TCGTATGCACAGTGCCGTATGTT |
| *RPS13* pre-mRNA R | TGATTTAGCGAACTATTCAATGCAACTTT |
| *RPS13* mRNA F | TCGTATGCACAGTGCCGGTAA |
| *RPS13* mRNA R | AGGACAACTTGAACCAAGCTGGAG |
| *ALG9*_F | TAAGCTGGCATGTGCTGCATTC |
| *ALG9*_R | TTTGCATGATTCGGTTGATTGG |
| *TIF3*_F | ATTGGAGTAGTGCTAGAGGCTCC |
| *TIF3*_R | GGCTTACCAAACTGAGCACCTC |
| *EGT2*_F | ATGTACTGAGTCTGGGCAAGCT |
| *EGT2*_R | TGTGCTGGAACTTGTTGGCA |
| *EST1*_F | CACATGCCACCATAGATAATGG |
| *EST1_*R | CGCATGATATCATGGAACCAGTGC |
| *PUS7_*F | CAACGTACTCAGGCAGATGGT |
| *PUS7*_R | AACCCTTTGGCACGTTACTGC |
| *PEX4*_F | ATGCCAAACTTCTGGATTCTTG |
| *PEX4*_R | TCAATGGTTGTTGATCCGCTCT |
| *NOP1*_F | TCAGACCAGGTAGCAGAGGTG |
| *NOP1*_R | TGATTCACCTGGGGCCATGTT |
| *RPS6B*_F | AGACGACTGAGCCATCATGAAG |
| *RPS6B_*R | TCCCTGCTGTCCTTTCTTCCG |
| *RPL13B*_F | AAGCAGGAATCAAGTAACAATGG |
| *RPL13B*_R | CCTTTTAGTGCAACCCGCCTA |
| *RPL22A*_F | ATCCACGCCCGATTACGAAG |
| *RPL22A*_R | CAAGTTACCGACAGCACCTTCG |
| *RPL26B_*F | GCTAAATAGTCGCCCTATGCCT |
| *RPL26B*_R | GTGAGGTGTGGATGTATAGAGG |

**Supplemental Table S3.**

Oligonucleotides used for RT-qPCR or RT-PCR to determine splicing status:
