# Supplementary material for: Transcription rate strongly affects splicing fidelity and cotranscriptionality in budding yeast
Source: Genome Res. 2018 Feb;28(2):203–13. doi: 10.1101/gr.225615.117 (PMC5793784; doi:10.1101/gr.225615.117)
Supplement: Supplemental Material [file supp_gr.225615.117_Supplemental_Figures.pdf]

Supplemental Figures

Transcription Rate Strongly Affects Splicing Fidelity and Co-transcriptionality in Budding Yeast

Vahid Aslanzadeh[1,3], Yuanhua Huang[2,4], Guido Sanguinetti[2] and Jean D. Beggs[1*]

1. Wellcome Centre for Cell Biology, University of Edinburgh, Michael Swann Building, Kings Buildings, Mayfield Road, Edinburgh EH9 3BF, UK
2. School of Informatics, University of Edinburgh, 10 Crichton St, Edinburgh EH8 9AB, UK
3. Current address: MRC Human Genetics Unit, Institute of Genetics and Molecular Medicine, University of Edinburgh, Crewe Road, Edinburgh EH4 2XU, UK
4. Current address: European Bioinformatics Institute, Wellcome Genome Campus, CB10 1SD, UK

***** Correspondence to: e-mail: jbeggs@ed.ac.uk

Contents

Supplemental Figure S1: Growth rate of the fast and slow RNAPII mutant strains

Supplemental Figure S2: Effects of transcription elongation on splicing efficiency

Supplemental Figure S3: Position distribution of all novel alternative splice sites

Supplemental Figure S4: Sashimi plots

Supplemental Figure S5: RT-PCR analysis of 6 novel introns and 4 novel alternative splicing events

Supplemental Figure S6: Features associated with splicing error rate and the density of downstream AG dinucleotides

Supplemental Figure S7: SEF distribution of longer isoforms versus shorter isoforms; Correlation between splicing fidelity and intron delta G; *FES1* sashimi plot

Supplemental Figure S8: Frequency of exon skipping in transcripts with two introns

Supplemental Figure S9: Yield of newly synthesised 4tU-labelled RNA

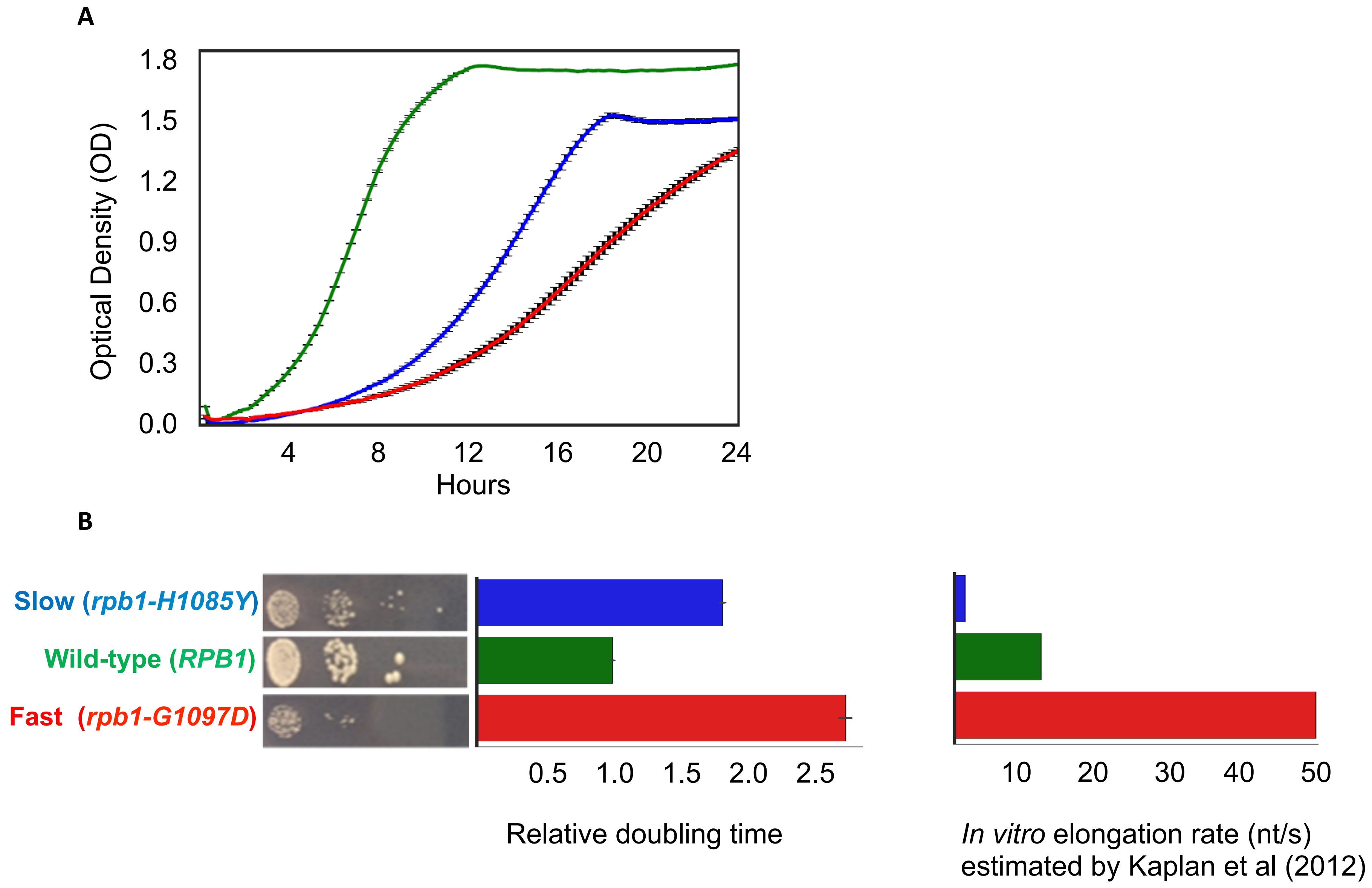

Supplemental Figure S1. Growth rate and estimated *in vitro* elongation rate of the fast and slow RNAPII mutants. (*A*) Growth curve of the RNAPII mutants and WT strain measured using Tecan Sunrise microplate reader in SC-LEU (synthetic complete growth medium lacking leucine). Error bars represent two biological replicates. (*B*) Spotting assay shows growth on SC-LEU after 3 days. Cell doubling time shown as barplot estimated from growth assay in liquid medium and normalized to the WT. In vitro elongation rate (right), nucleotide per second (nt/s), quantified by Kaplan et al (2012) using transcription run-off assay.

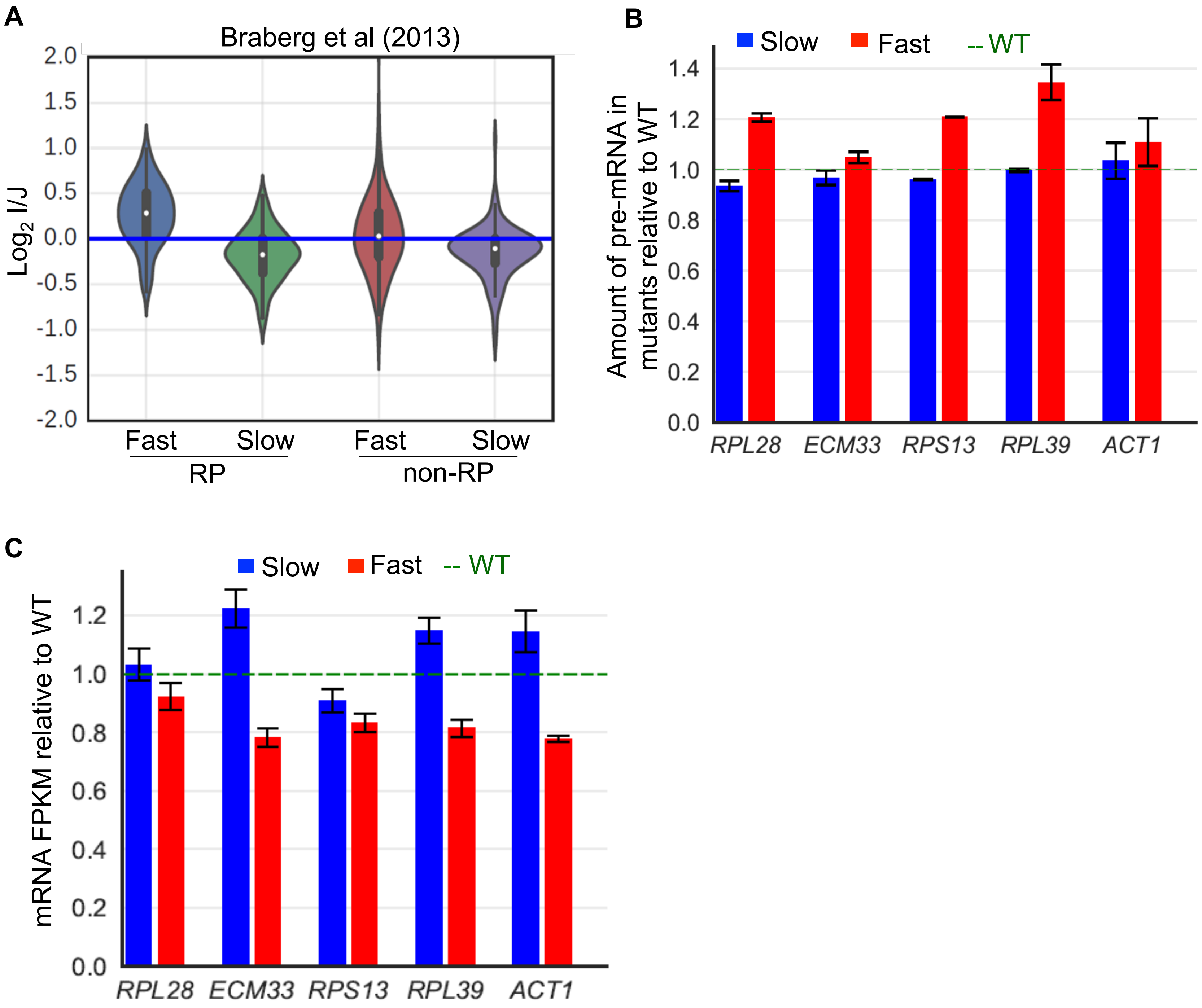

Supplemental Figure S2. (*A*) Splicing efficiency of RP and nonRP transcripts measured by splicing-sensitive microarrays. Data are from Braberg et al (2013). The fast mutant is *rpb1-G1097D* and the slow mutant is *rpb1-H1085Q*. *Y axis* shows I (Intron) over J (Junction) ratio in $\log_2$ scale normalized to wild type. Horizontal blue line in *y axis* is WT level. (*B*) Amount of pre-mRNA and (*C*) mRNA levels for 5 example genes measured by DICEseq from RNA sequencing data.

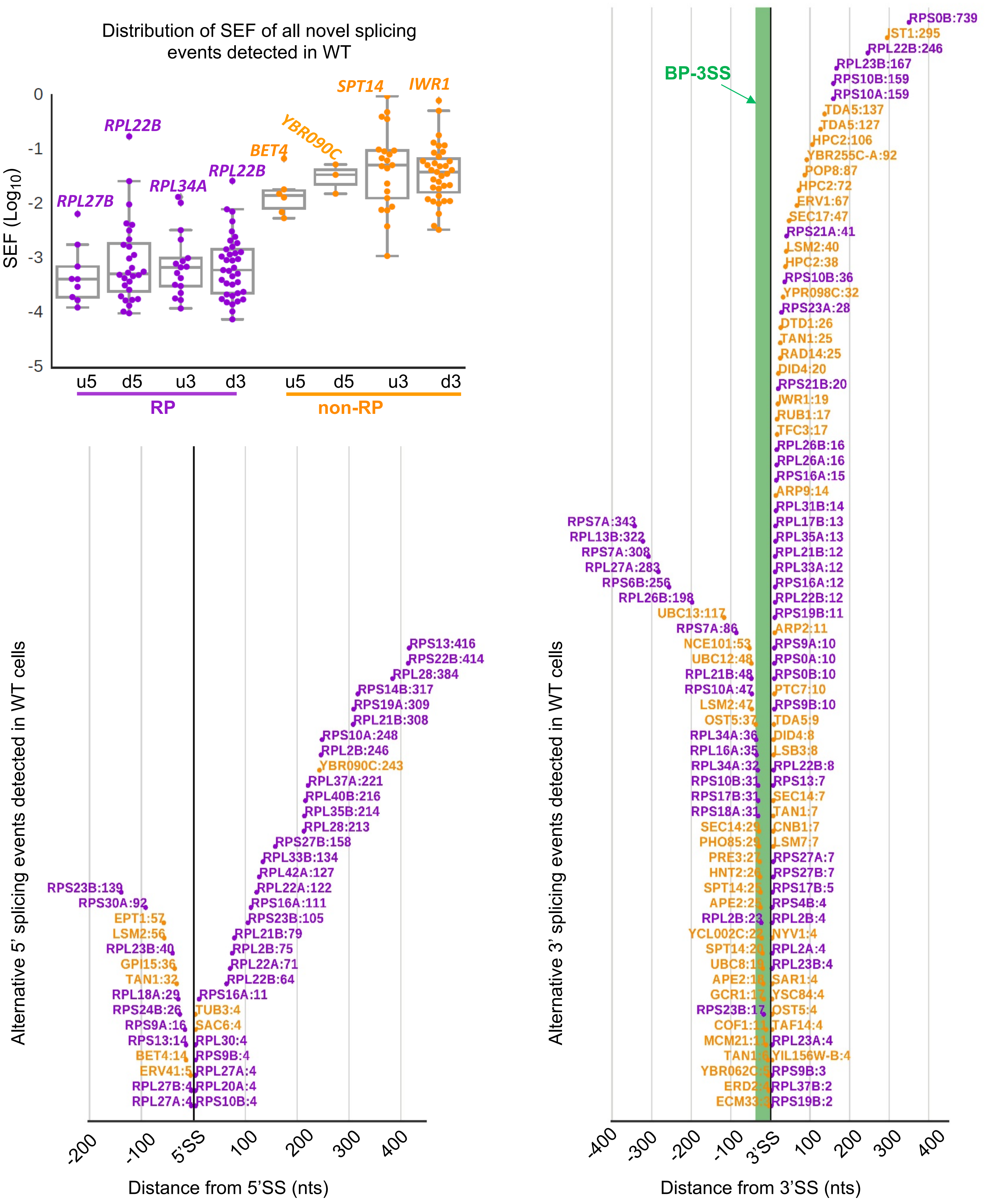

Supplemental Figure S3. Position distribution of all novel alternative splice sites from annotated 5'SSs (*left*) and 3'SSs (*right*) in RP (*purple*) and non-RP (*orange*) intron-containing transcripts in WT. d3 is downstream and u3 is upstream relative to the annotated 3'SS, and d5 is downstream and u5 is upstream relative to the annotated 5'SS. Number indicates the distance in nucleotides. Green region upstream of 3'SS is the mean distance between BP and annotated 3'SS in budding yeast (~37 nucleotides). Boxplot (upper left) shows SEF for all novel alternative splicing events in RPs and non-RPs in WT. Events with highest SEF are indicated with their gene names.

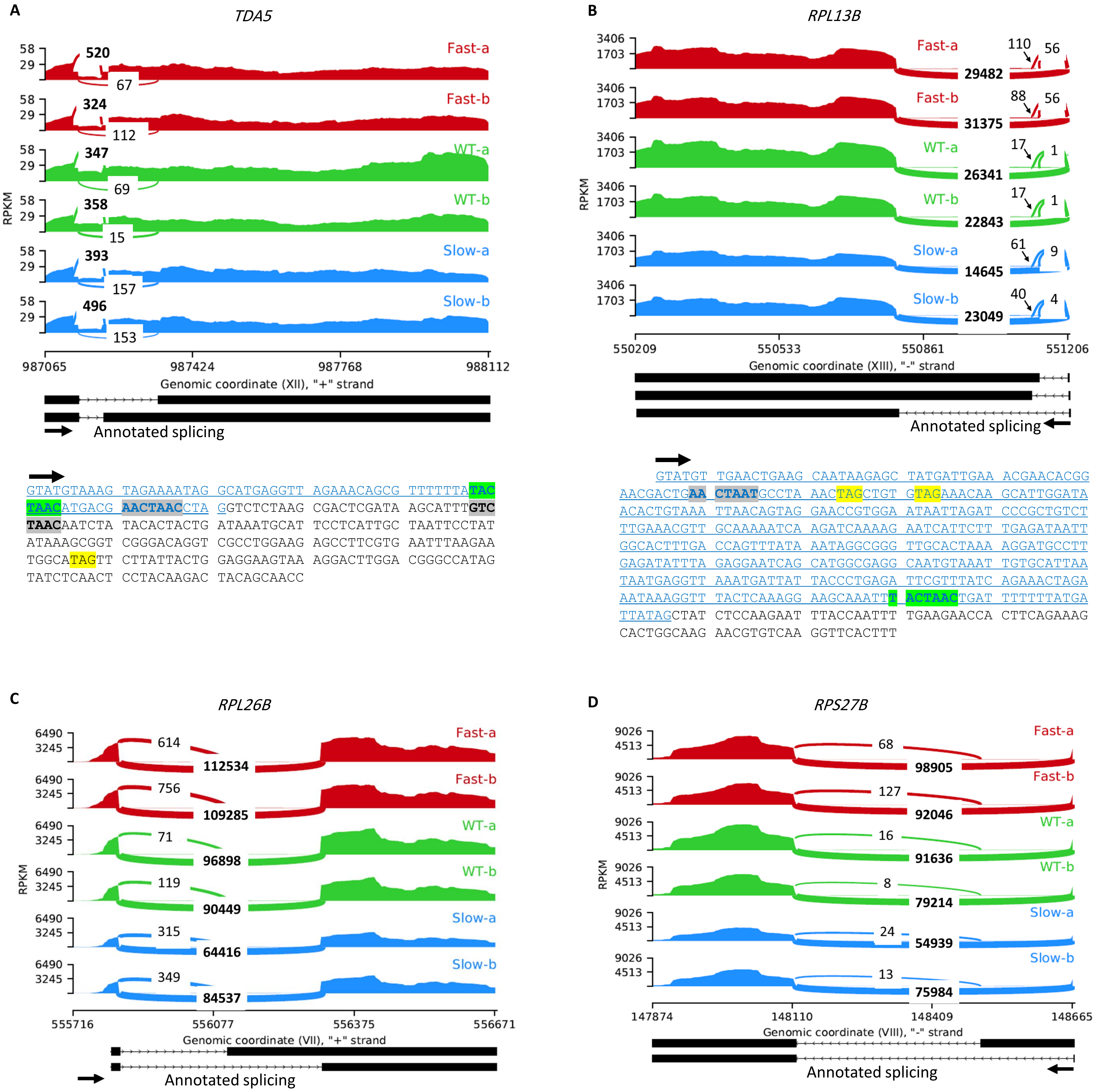

A
TDA5
Fast-a
520
67
Fast-b
324
112
WT-a
347
69
WT-b
358
15
Slow-a
393
157
Slow-b
496
153
58
29
RPKM
987065
987424
987768
988112
Genomic coordinate (XII), "+" strand
Annotated splicing
GTATGTAAAG TAGAAAATAG GCATGAGGTT AGAAACAGCG TTTTTTATAC
TAACATGACG AACTAACCTA GGTCTCTAAG CGACTCGATA AGCATTTGTC
TAACAATCTA TACACTACTG ATAAATGCAT TCCTCATTGC TAATTCCTAT
ATAAAGCGGT CGGGACAGGT CGCCTGGAAG AGCCTTCGTG AATTTAAGAA
TGGCATAGTT CTTATTACTG GAGGAAGTAA AGGACTTGGA CGGGCCATAG
TATCTCAACT CCTACAAGAC TACAGCAACC
B
RPL13B
Fast-a
110
56
29482
Fast-b
88
56
31375
WT-a
17
1
26341
WT-b
17
1
22843
Slow-a
61
9
14645
Slow-b
40
4
23049
3406
1703
RPKM
550209
550533
550861
551206
Genomic coordinate (XIII), "-" strand
Annotated splicing
GTATGT TGAACTGAAG CAATAAGAGC TATGATTGAA ACGAACACGG
AACGACTGAA CTAATGCCTA AACTAGCTGT GTAGAAACAA GCATTGGATA
ACACTGTAAA TTAACAGTAG GAACCGTGGA ATAATTAGAT CCCGCTGTCT
TTGAAACGTT GCAAAAATCA AGATCAAAAG AATCATTCTT TGAGATAATT
GGCACTTTGA CCAGTTTATA AATAGGCGGG TTGCACTAAA AGGATGCCTT
GAGATATTTA GAGGAATCAG CATGGCGAGG CAATGTAAAT TGTGCATTAA
TAATGAGGTT AAATGATTAT TACCCTGAGA TTCGTTTATC AGAAACTAGA
AATAAAGGTT TACTCAAAGG AAGCAAATTT ACTAACTGAT TTTTTTATGA
TTATAGCTAT CTCCAAGAAT TTACCAATTT TGAAGAACCA CTTCAGAAAG
CACTGGCAAG AACGTGTCAA GGTTCACTTT
C
RPL26B
Fast-a
614
112534
Fast-b
756
109285
WT-a
71
96898
WT-b
119
90449
Slow-a
315
64416
Slow-b
349
84537
6490
3245
RPKM
555716
556077
556375
556671
Genomic coordinate (VII), "+" strand
Annotated splicing
D
RPS27B
Fast-a
68
98905
Fast-b
127
92046
WT-a
16
91636
WT-b
8
79214
Slow-a
24
54939
Slow-b
13
75984
9026
4513
RPKM
147874
148110
148409
148665
Genomic coordinate (VIII), "-" strand
Annotated splicing

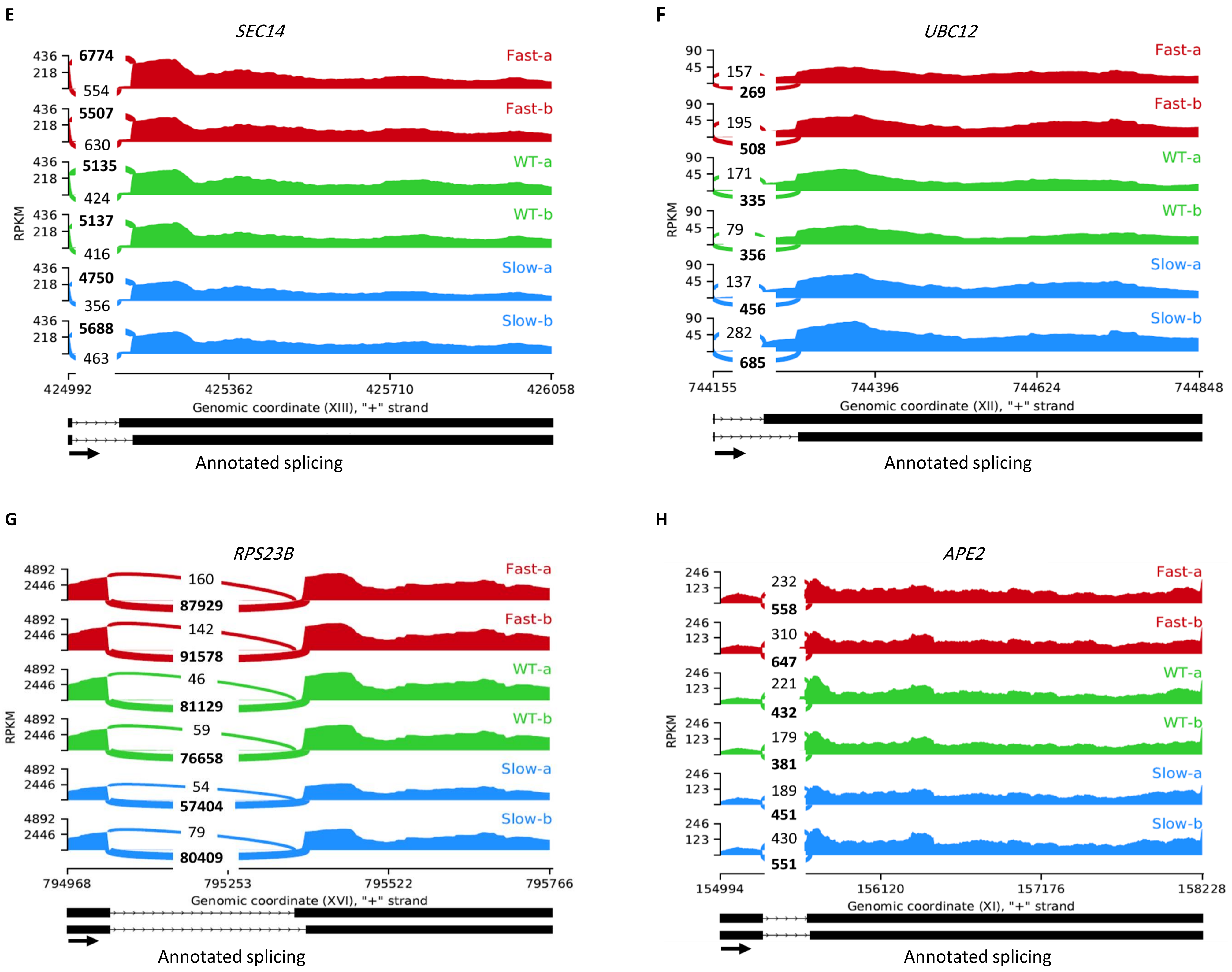

Supplemental Figure S4. Sashimi plots were generated with the MISO package (Katz et al. 2010). Arcs represent junction reads connecting first and second exon borders. Resulting isoforms are drawn at the bottom of the plot. *Y axis* shows Reads Per Kilobase of transcript per Million mapped reads (RPKM) in each sample. Numbers bold font represent read counts for the annotated junctions. (*A*) Position of the cryptic BPs and novel 3'SSs (127 nts downstream) in *TDA5*. (*B*) Position of the cryptic BP and alternative 3'SSs (332 and 330 nucleotides upstream) at the 5'end of the intron in *RPL13B*. Annotated BPs are highlighted with green, cryptic BPs identified with lariat sequencing (Gould et al. 2016) are highlighted with grey, intron sequence is shown in blue, novel splice sites are highlighted with yellow, partial sequence from exon2 is shown in black. (*C*) Alternative 3'SS (198 nts upstream) in *RPL26B*. (*D*) Alternative 5'SS (158 nts downstream) in *RPS27B.* (*E*) Alternative 3'SS (29 nts upstream) in *SEC14.* (*F*) Alternative 3'SS (48 nts upstream) in *UBC12.* (*G*) Alternative 3'SS (17 nts upstream) in *RPS23B.* (*H*) Alternative 3'SS (18 nts upstream) in *APE2.*

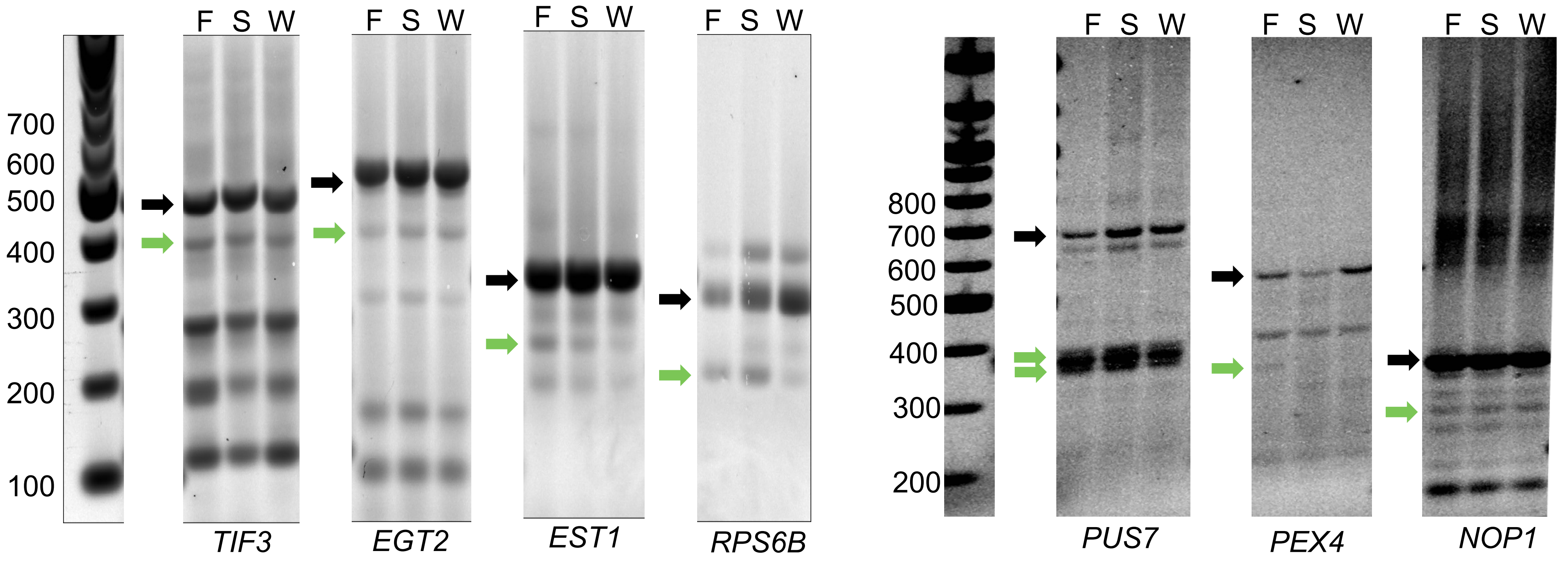

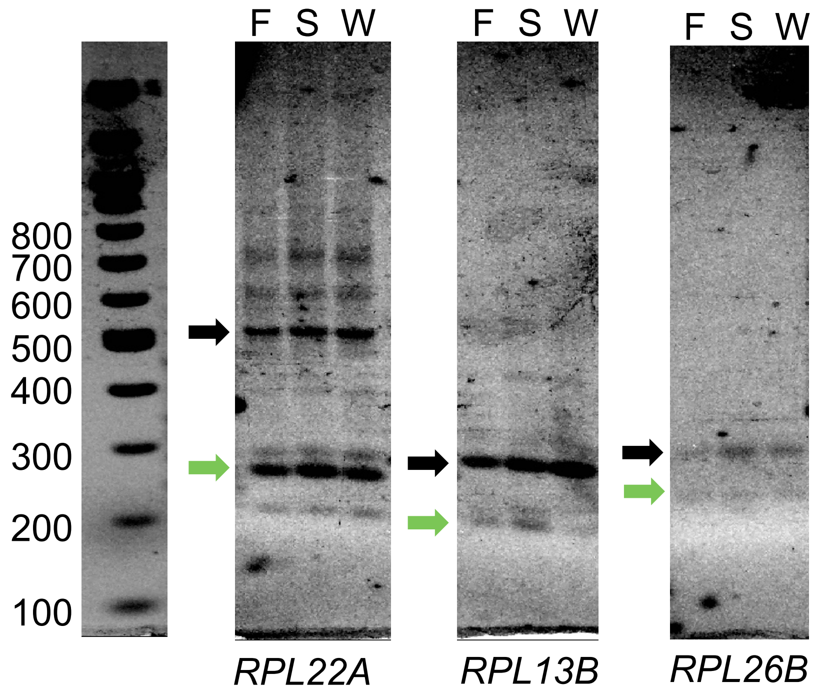

PCR product (unspliced)	PCR product (spliced)	Chr	Gene	Intron Start	Intron end	Event
484	407	XVI	TIF3	869107	869184	newintron
525	412	XIV	EGT2	26746	26859	newintron
338	245	XII	EST1	609403	609496	newintron
705	366,383	XV	PUS7	791693	792015	newintron
552	348	VII	PEX4	756948	757152	newintron
341	288	IV	NOP1	427467	427520	newintron
309	211	II	RPS6B	592673	592768	u3
262	190	XIII	RPL13B	551132	551203	u3
508	241	XII	RPL22A	263328	263594	d5
283	203	VII	RPL26B	555831	555910	u3

Supplemental Figure S5. RT-PCR analysis of 6 novel introns in *TIF3*, *EGT2*, *EST1*, PUS7, *PEX4* and *NOP1*, and 4 novel alternative splicing events observed for *RPS6B*, *RPL22A*, *RPL13B* and *RPL26B*. The expected position of the PCR products confirming spliced and unspliced species are marked with green and black arrows, respectively. F, S and W denote Fast, Slow and Wild Type. Table shows expected size of spliced and unspliced PCR products and novel intron coordinates on the genome. PCR product of novel splicing event for *PEX4* is only observed with the fast mutant which confirms RNA-seq data that only has reads for this event in the fast mutant with SEF = 0.026. RNA sequencing detects novel splicing for *EST1* in the fast and slow mutants with SEF=0.017 and SEF=0.0085, respectively, and zero reads for this event in WT cells. Accordingly, intensity of spliced product is stronger in the fast and slow mutants but a faint spliced PCR product is detected in WT cells. Alternative upstream 3'SS for *RPS6B* also occurs with higher frequency in the fast (SEF=0.0013) and slow (SEF=0.0007) mutants compared to WT (SEF=0.0002). Relative intensity of spliced vs unspliced PCR products confirms what we observe with RNA-seq. Additionally, use of alternative upstream 3'SS for *RPL13B* is increased with the fast (SEF=0.0018) and slow (SEF=0.0003) mutants but there were no reads supporting occurrence of this event in WT.

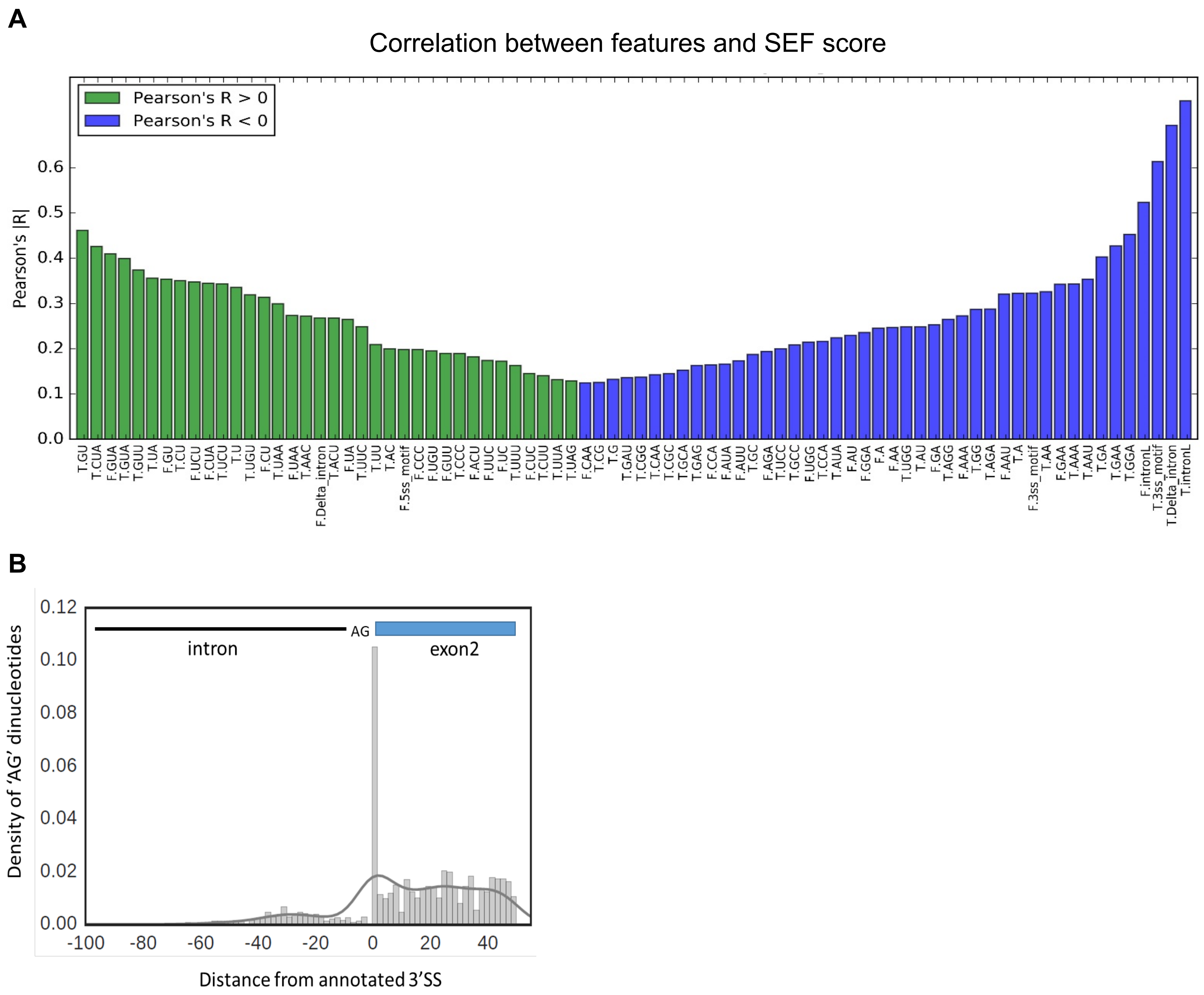

Supplemental Figure S6. (*A*) Features associated with splicing error rate. Pearson's correlations between splicing fidelity and sequence patterns shows the significantly correlated features ($P < 0.05$) to SEF. *Green* represents positive correlation and *blue* represents negative correlation. "T" and "F" prefixes are used to show whether features come from annotated or novel introns, respectively. (*B*) Density of 'AG' dinucleotides in the region between annotated BP positions in all intron-containing genes and 50 nucleotides into exon2 of all intron-containing transcripts. The highest density belongs to the 'AG' of the annotated 3'SS.

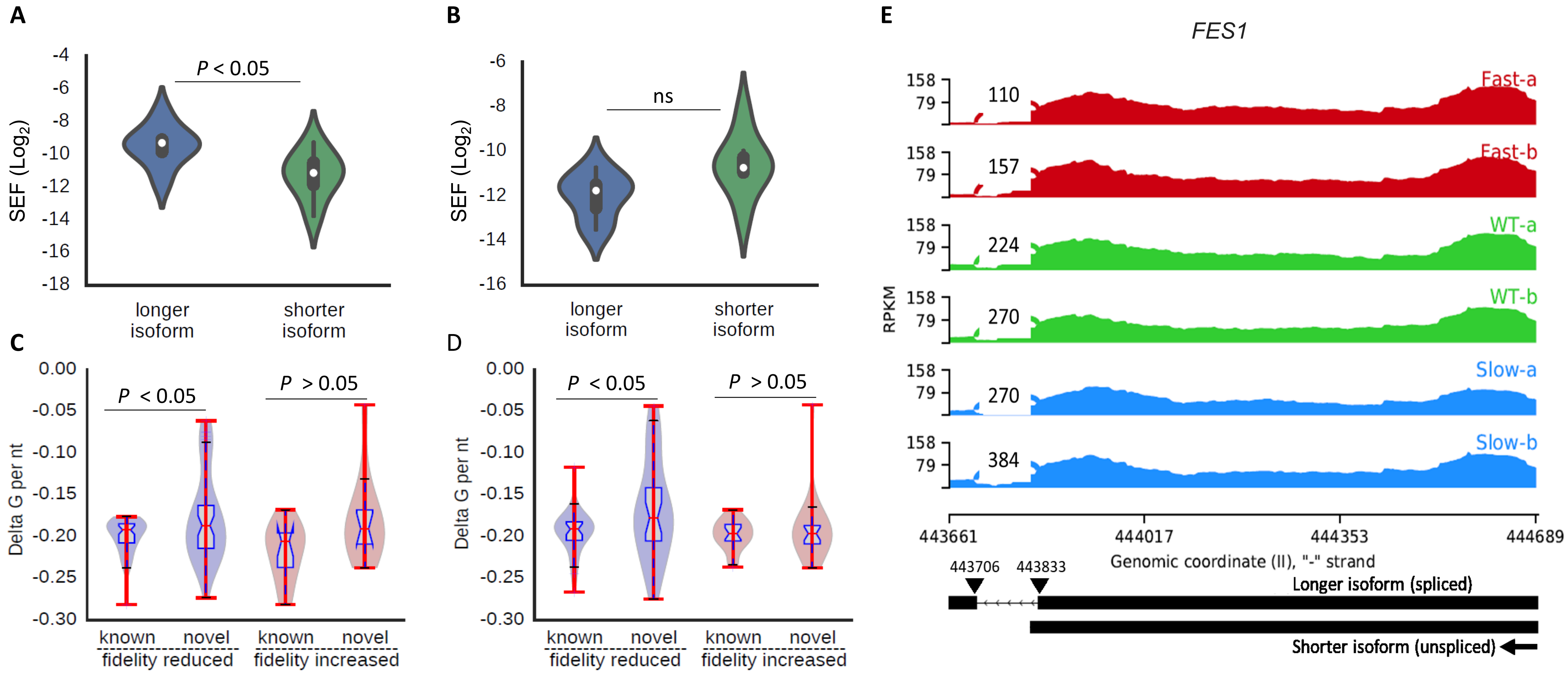

Supplemental Figure S7. (*A*) SEF distribution of longer isoform vs shorter isoform in 7 RP paralogs (*RPL16B*:*RPL16A*, *RPL27A*:*RPL27B*, *RPL43A*:*RPL43B*, *RPL2B*:*RPL2A*, *RPS27B*:*RPS27A*, *RPS21B*:*RPS21A*, *RPS19B*:*RPS19A*) with highest intron length difference between two isoforms. Differences between shorter and longer isoforms distributed between 138nts up to 253nts. (*B*) SEF distribution of longer isoform vs shorter isoform in 7 RP paralogs (*RPL33B*:*RPL33A*, *RPL17B*:*RPL17A*, *RPS10A*:*RPS10B*, *RPL26B*:*RPL26A*, *RPL37B*:*RPL37A*, *RPL23A*:*RPL23B*, *RPS18A*:*RPS18B*) with lowest intron length difference between two isoforms. Differences between shorter and longer isoforms distributed between 2nts up to 34nts. (*C-D*) Correlation between splicing fidelity in fast (left panel) and slow (right panel) mutants and intron delta G per nucleotide of known and novel RP introns. The *P-values* were obtained by t-test. (*E*) *FES1* sashimi plot. Arcs represent junction reads connecting first and second exon borders. Resulting isoforms are drawn at the bottom of the plot. *Y axis* shows Reads Per Kilobase of transcript per Million mapped reads (RPKM) in each sample.

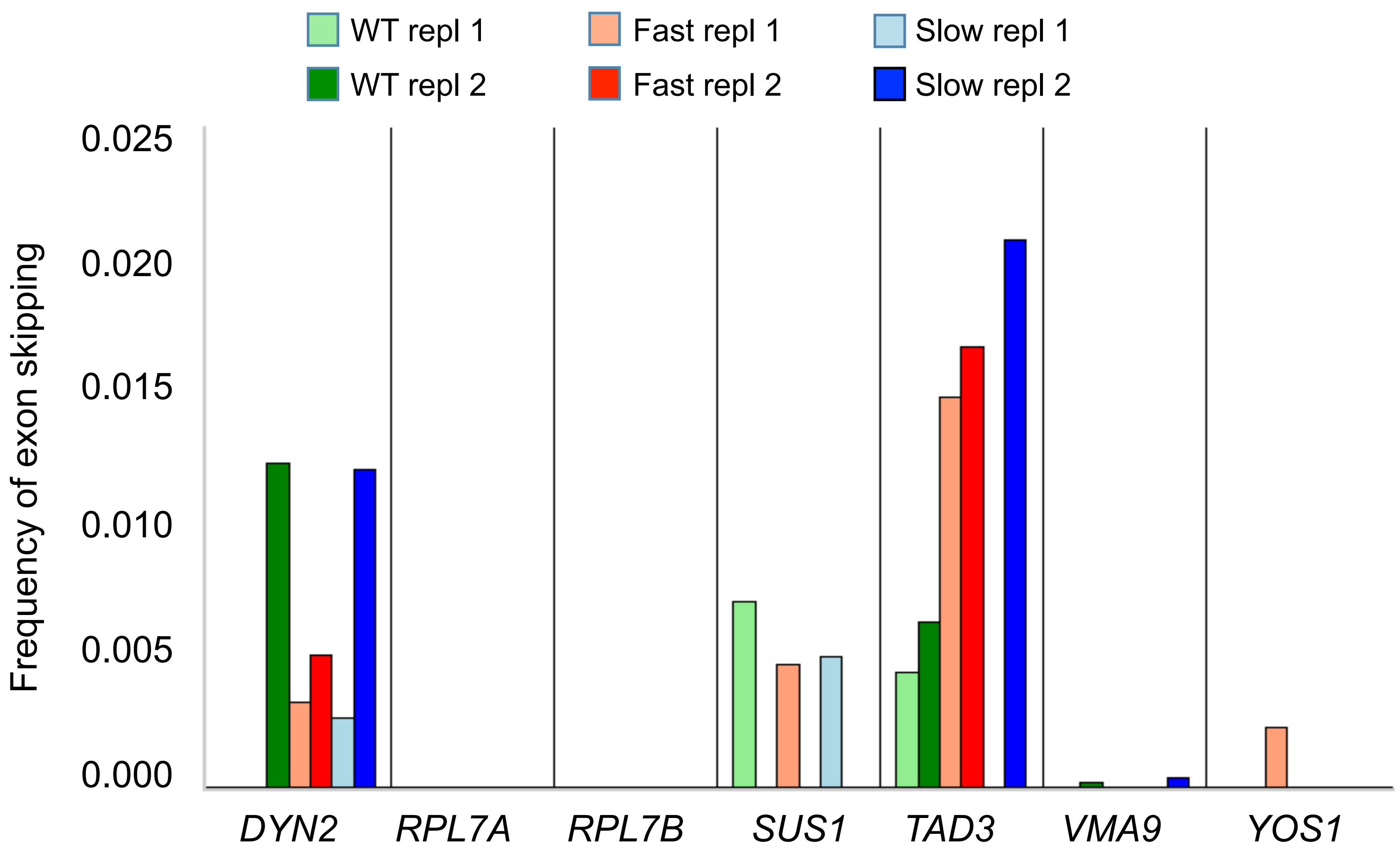

Supplemental Figure S8. Frequency of exon skipping in transcripts with two introns shown separately for both replicates. To calculate frequency of exon skipping, the number of exon1-exon3 junction (exon skipping) reads was divided by the number of exon1-exon2 junction (first intron splicing) reads. The absence of frequency bar for replicates in some genes indicates that reads supporting exon skipping are not detected in that replicate. For example, skipping reads not detected for *RPL7A* and *RPL7B* in both replicates of all three strains.

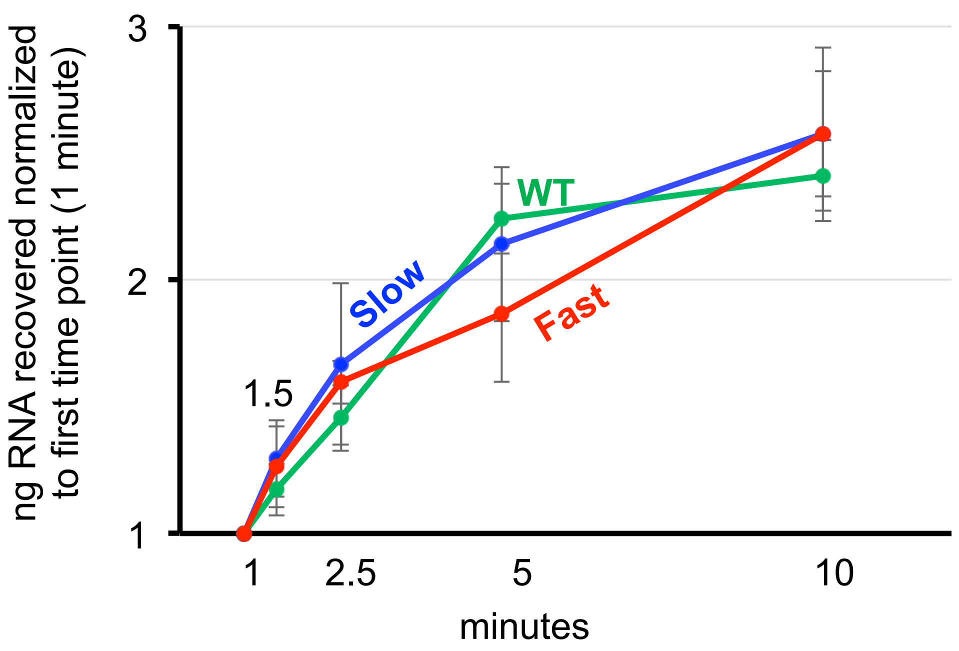

Supplemental Figure S9. Total yield of the newly synthesized RNA (4tU labelled) at the indicated times (at least three biological replicates). The amount of newly synthesized RNA increases with time in all strains.
