# Supplementary material for: Transcription rate strongly affects splicing fidelity and cotranscriptionality in budding yeast
Source: Genome Res. 2018 Feb;28(2):203–13. doi: 10.1101/gr.225615.117 (PMC5793784; doi:10.1101/gr.225615.117)
Supplement: Revised Supplemental Material [file supp_28_2_203_v2_index.html]

Revised Supplemental Material 

# Transcription Rate Strongly Affects Splicing Fidelity and Co-transcriptionality in Budding Yeast

## Revised Supplemental Material

- Supplemental\_Figures.pdf
